# Revealing Organophosphorus and Carbamate Interactions with Albumin Using ^1^H NOE Pumping NMR Technique

**DOI:** 10.1021/acs.analchem.5c02132

**Published:** 2026-01-28

**Authors:** Ivana V. Sofrenić, Sami Heikkinen, Anne Puustinen, Niko Minkkinen, Harri Kiljunen, Harri A. Heikkinen

**Affiliations:** † VERIFIN, Department of Chemistry, 3835 University of Helsinki, P.O. Box 55, FIN-00014 Helsinki, Finland; ‡ Faculty of Chemistry, 54801 University of Belgrade, Studentski trg 12-16, 11000 Belgrade, Serbia; § Department of Chemistry, University of Helsinki, P.O. Box 55, FIN-00014 Helsinki, Finland

## Abstract

In this work, the capability of the ^1^H nuclear Overhauser effect (NOE) pumping NMR technique was applied to elucidate the atomic-level binding interaction between the bovine serum albumin (BSA) and four toxic compounds: amiton, dimethoate, carbofuran, and aminostigmine. With the aid of ^1^H NOE pumping experiments, we were able to highlight ligand binding epitopes for the studied compounds and provide prefatory data for the ligand affinity with BSA via dissociation constant values (*K*_D_). In addition, we demonstrate that the ^1^H NOE pumping technique is suitable for the ligand competition studies solely using one NMR sample and that the technique is a simple and straightforward method capable of revealing important parameters that are used typically to define ligand–albumin interaction at the atomic level. We believe the novel precursory results herein provide important and experimentally driven data for the BSA interaction, especially for carbamate-based molecules, where the existing literature is fairly limited. Based on the preliminary experimental results, amiton and aminostigmine showed stronger binding to BSA based on NOE pumping data compared with dimethoate and carbofuran, although the obtained *K*_D_ values were observed within a similar range. Our results present the first comparable study between the organophosphorus (OP) and the carbamate (CM) toxic compounds with BSA via NMR spectroscopy only. Furthermore, the efficacy of the ^1^H NOE pumping technique provided evidence that the organophosphorus and carbamate compounds bind to a common epitope site on BSA.

## Graphical abstract

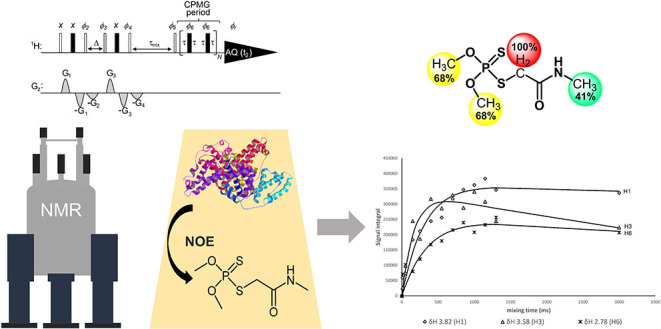

## Introduction

Various organophosphorus (OP)- and carbamate (CM)-based chemicals possess high toxicity toward mammals, and yet they are the two most widely used pesticides in the modern world.^1^ Since such pesticides are also widely used in agriculture for necessity, their poisonous nature against humans and other mammals is drawing attention. The misuse and indirect exposure of many toxic pesticides via water, food, and environment cause annually several intoxications for humans and animals as these chemicals may also be absorbed through the skin or lungs.^2,3^ Both OPs and CMs have the same basis for their mechanism of action causing toxicity: they are capable of inhibiting vital enzymes, acetylcholinesterase (AcChE) and butyrylcholinesterase (BuChE), either by phosphorylating (OPs) or by carbamylating (CMs) their active sites. Although the toxicity of OPs and CMs is mainly associated with AcChE inhibition, studies have shown that carcinogenic toxicity is also affected by the affinity to the serum proteins.^4^ Serum albumin, the predominant plasma protein, facilitates systemic transport of drugs via molecular interactions, enabling regulated biodistribution through the circulation.^5^ Free forms of compounds can cross biological membranes and interact with target enzymes, such as AcChE in the case of pesticides, to exert poisonous effects. Albumin acts as a reservoir, binding to a significant portion of the compound and reducing the concentration of the free active form. The binding capacity of albumin buffers against high concentrations of the free compound, thus mitigating toxicity.^6^ Therefore, in the case of OP and CM, stronger ligand binding to albumin leads to lower toxicity via lesser AcChE inhibition capacity.

Typically, ligand–protein interactions are investigated with fluorescence spectroscopy,^7^ isothermal titration calorimetry,^8^ and circular dichroism.^9^ The existing literature for experimental data for pesticide–protein interactions is rather limited. Chemical analytical methods used to study the OP pesticide–protein and OP metabolite–protein interactions include NMR spectroscopy,^10,11^ isothermal calorimetry (ITC),^10^ UV–vis spectroscopy,^12,13^ circular dichroism (CD), and FT-IR spectroscopy.^14^ The study by Dahiya et al. highlighted the binding interactions for chlorpyrifos, diazinon, and parathion with bovine serum albumin (BSA) using ^1^H saturation transfer difference (STD) NMR, where similar binding modes for all three OPs were observed, primarily involving the aryl ring of the compounds.^10^ Binding constants with varying strengths of interactions between the studied pesticides and BSA were also reported. The research by Chaubey and Pal utilized ^19^F NMR to demonstrate how organofluorine compounds interact with serum albumin.^11^

NMR spectroscopy is a widely utilized technique for studying small-molecule binding with proteins or other macromolecules. Traditionally, two common NMR approaches are used: either by directly observing changes in the protein structure via chemical shift perturbation with ligand titration or by observing the ligand’s signal alteration upon ligand binding via the nuclear Overhauser effect (NOE). Depending on the situation, the ligand-observed approach is often chosen in chemical biology and pharmaceutical research areas as this method does not require any isotopically labeled proteins, is not limited to protein size, is a more economically viable method, and does not require special NMR instrumentation.^15,16^ Typical, NOE-dependent, ligand-observed NMR methods are saturation transfer difference (STD NMR),^17^ exchange-transferred NOE (et-NOESY),^18^ and Water Ligand Observed via Gradient SpectroscopY (waterLOGSY).^19^ The ^1^H NOE pumping method, originally developed by Shen and Shapiro,^20,21^ is perhaps a less recognized method as it has been applied previously in other types of interaction studies, such as NMR chemosensing.^22^ Interestingly, all of the other methods (STD NMR, waterLOGSY, and et-NOESY) rely on observing the NMR parameters between the free and the bound states of the molecules, whereas the NOE pumping method relies on the molecule’s free translational diffusion ability in the given solvent. In fact, we used ^1^H NOE pumping method in our study as we believe that the method may have a few advantages over the other NOE-based methods: no spectral subtraction is required compared with STD NMR (subtraction artifacts originating, e.g., from pH changes are excluded), all of the detected signals are in the same phase compared with waterLOGSY, and the signals are observed with one-dimensional NMR spectra compared with 2D et-NOESY (faster acquisitions). In ^1^H NOE pumping, differences in the free translational molecular diffusion of the protein and the ligand are utilized to suppress the signals of fast diffusing molecules, ideally leaving only protein signals. During the mixing time, NOE interaction between the protons of proteins and weakly binding ligands create the observable signals of interacting ligand protons. Obviously, the protein signals will also be observable after NOE mixing, but these can be readily suppressed by a T_2_-filter prior to acquisition period. In the case of strong ligand binding, i.e., in the micromolar range or stronger, the diffusion filter does not eliminate these ligand signals, and they would be observable even without NOE mixing, and thus a series of spectra with increasing mixing times does not follow the expected NOE build-up behavior. Therefore, NOE pumping is not suitable for studying strong binding interactions.

Despite the existing pesticide–protein interaction data, the available experimentally driven atomic-level information is limited, especially for carbamate-based pesticides. This underscores the necessity for further research into the unique characteristics and implications of carbamate toxicity within the context of chemical safety and regulatory frameworks. This was mainly the reason we wanted to include both types, OP- and CM-based ligands, in our study. The studied compounds, dimethoate (OP pesticide, toxicity class II), carbofuran (CM pesticide, toxicity class Ib), amiton (OP nerve agent, OPCW Schedule II), and aminostigmine (CM, nerve agent precursor in OPCW Schedule I) are all classified as highly toxic compounds (Figure 1). Whereas dimethoate and carbofuran are commonly used insecticides and acaricides in the modern world, amiton has been considered too dangerous for agricultural purposes and is classified as a nerve agent under Schedule 2 of the Chemical Weapons Convention (CWC). In 2020, significant modifications were made to the list of scheduled compounds under the CWC, marking the first inclusion of four new cholinesterase inhibitors, known as nerve agents.^23^ Among them, carbamates garnered considerable attention due to the expectation of having distinct properties compared with the OP-type nerve agents. Despite their relevance, there exists a paucity of literature data regarding the toxicological effects of these compounds, highlighting a critical gap in our understanding of their potential risks and mechanisms of action. In fact, as can be seen from Figure 1, nerve agents resemble pesticides in a way that their classification or toxic nature toward human beings cannot be instantly drawn based on the chemical structure alone.

**Figure 1:**
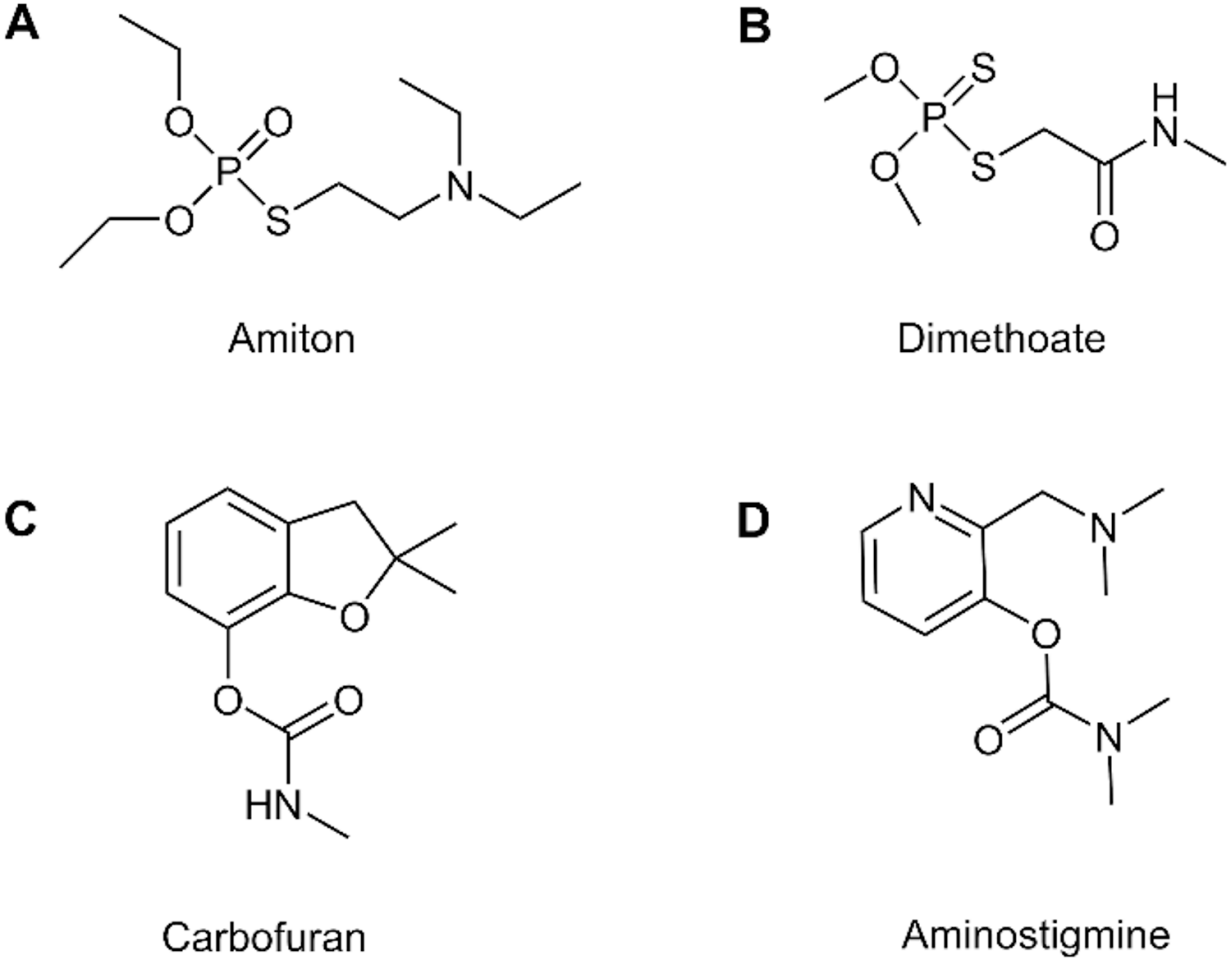
Chemical structures of (A) amiton (OP nerve agent, CAS number 78–53–5), (B) dimethoate (OP pesticide, CAS number 60–51–5), (C) carbofuran (CM pesticide, CAS number 1563–66–2), and (D) aminostigmine (CM nerve agent precursor, CAS number 67049–84–7).

The main goal of this study was to discover how the OP- and CM-based molecules differ upon their interaction with the serum albumin and to show the capabilities of the ^1^H NOE pumping method for investigating toxic ligand binding with BSA at the atomic level. BSA was chosen as a model protein over human serum albumin (HSA) as it was obtained at low cost and is structurally similar to HSA. The BSA protein (66.5 kDa) structure is composed of three different subdomains (I, II, and III), which all are divided further into two different subdomains (A and B). Ligands are known to bind with BSA sites located in the hydrophobic cavities in subdomains IIA and IIIA.^24^

## Experimental Section

### Materials

The bovine serum albumin (BSA, purity 98%) was purchased from Fluka Biomedica and used without further purification. Dimethoate, carbofuran, d-glucose, and TSP-*d4* were purchased from Sigma-Aldrich. Amiton and aminostigmine were synthesized in-house, and the structures were verified with NMR and LC-MS (NOTE! Amiton and aminostigmine are highly toxic compounds and should be handled by trained professionals only). The purity of the in-house synthesis products was measured using TraceCERT chemicals durene [CAS 95–93–2] and maleic acid [CAS 110–16–7] as internal standards. Deuterium oxide (D_2_O, 99.9% D, Euroisotop) and acetonitrile-*d3* (ACN-*d3*, 99.8% D, Euroisotop) were used as the solvents. All used chemicals were of analytical grade. The stock solutions of d-glucose, dimethoate, and amiton were prepared in D_2_O, and carbofuran and aminostigmine in ACN-*d3*. The working stock solutions were prepared using phosphate buffer pH 7.4 in D_2_O in order to minimize the concentration of acetonitrile and prevent effects on BSA structure.

### Sample Preparation

Final concentrations in the NMR tube were 50 μM BSA, 5.0 mM d-glucose, and 5.0 mM tested compound in a total volume of 600 μL, maintaining a BSA:ligand ratio of 1:100 for ligand binding epitope mapping experiments. The same conditions were applied for the three-component system. The solutions used for NMR experiments in BSA–ligand titration studies contained 50 μM BSA, 5 mM d-glucose, and a varying concentration of ligand ranging from 1.5 to 15.0 mM, maintaining BSA:L ratios of 1:30, 1:50, 1:70, 1:100, 1:120, 1:150, 1:200, and 1:300.

### NMR Spectroscopy

All NMR experiments were measured using a Bruker Avance III 500 NMR spectrometer (^1^H operating frequency of 500.03 MHz) equipped with a 5.0 mm inverse-detection triple-resonance TXI probe head (^1^H, ^13^C, ^31^P) incorporating a *z*-axis gradient coil capable of delivering gradient amplitudes up to 50 G/cm. The ^1^H-pulse flip-angle calibration (Bruker macro “pulsecal”) was executed prior to each measurement. Chemical shifts were reported against TSP-*d4* (3-(trimethylsilyl)-2,2,3,3-tetradeuteropropionic acid, δ_1H_ 0.00 ppm). Topspin 3.5 pl7 software (Bruker, Germany) was used to acquire and process the NMR spectra.

The chemical structures and concentrations of the ligands used in this study were confirmed with typical NMR experiments (quantitative ^1^H, ^13^C­{^1^H}, 2D-[^1^H–^1^H]-COSY, 2D-[^1^H–^1^H]-NOESY, 2D-[^1^H–^13^C]-HSQC, 2D-[^1^H–^13^C]-HMBC, 2D-[^1^H–^31^P]-HMQC, and ^31^P­{^1^H}). The quantitative ^1^H NMR experiment was used to calculate the exact ligand concentration utilizing TraceCERT chemicals (durene and maleic acid) as internal standards. Information regarding the ligand concentration and purity profile was important to complete the calculations needed for the correct molar ratios used in the ^1^H NOE pumping experiments. Assigned NMR spectra together with the obtained purity of the ligands can be found in the Supporting Information (SI). Multiplicity is given as follows: s = singlet, d = doublet, t = triplet, q = quartet, m = multiplet, and br = broad signal.

### ^1^H NOE Pumping Experiment

^1^H NOE pumping experiments were measured with a slightly modified pulse sequence originally developed and described by Chen and Shapiro.^21^ The standard Bruker diffusion pulse sequence BPPLED (Bipolar Pulse Pair Longitudinal Eddy current Delay, ledbpgp2s1d)^25^ was modified to include a CPMG-based^26,27^ T_2_-filter prior to the acquisition period to remove the unwanted protein signals. In addition, gradient blanking (and the activation of field-frequency lock) was applied after the eddy current recovery delay following gradient pulse G_4_. Solvent suppression was achieved with a simple gated presaturation period during the relaxation delay (d_1_) and was used when needed. If not stated otherwise, the typical acquisition parameters were as follows: number of scans (ns) 128, spectral width (sw) 15 ppm, 7500 Hz, number of points (td) 64k, acquisition time (aq) 4.4 s, relaxation delay (d_1_) 15 s, number of dummy scans (ds) 32, diffusion gradient duration (G_1_ and G_3_) 1 ms, diffusion gradient strength (smoothed square, SMSQ10.50) 95% (47.5 G/cm), diffusion time (Δ) 250 ms, and NOE mixing time (*t*_mix_) 10–3000 ms resulting in average to 45 min per experiment varying mainly on the used mixing time. All NOE pumping experiments were measured at 300 K. In addition, a comparable amount of glucose with the ligand was added to each NMR sample to operate as a negative control (i.e., no signals from the glucose should be visible in the final NOE pumping spectrum). The authors are aware of the binding of TSP-*d4* toward proteins, and hence the signal from TSP-*d4* served as a positive control in the used method. The utilized pulse sequence (Figure 2) is provided in SI and is available upon request from the authors.

**Figure 2:**
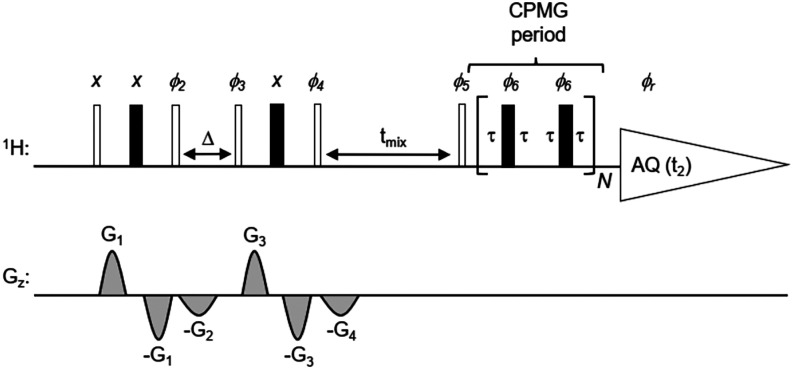
Pulse sequence used for the ^1^H NOE pumping experiments. This pulse sequence is essentially a BPPLED (Bipolar Pulse Pair Longitudinal Eddy current Delay)^28^ experiment including a T_2_-filter (CPMG block prior to acquisition) for suppressing macromolecule signals. Gradient blanking and the activation of the field-frequency lock were performed after the eddy current recovery delay for gradient pulse G_4_. Solvent presaturation was used during the relaxation delay when needed. *Pulses*: Narrow (wide) bars correspond to 90° (180°) hard rectangular pulses. Gradient pulses are represented by gray shapes denoted by G_1_–G_4_. G_1_ and G_3_ are diffusion gradients (1000 μs), and G_2_ and G_4_ are homospoil gradients (600 μs). *Delays*: Δ = diffusion time (250 ms), *t*_mix_ = mixing time for NOE build-up (10–3000 ms), and τ = CPMG delay (242 μs). Total duration of the CPMG period is 4*Nτ*, where *N* is the loop counter. Typical CPMG durations range from 50 to 100 ms. Phase cycle: ϕ_2_ *= x x −x −x; ϕ*_3_ *= x x x x −x −x −x −x y y y y −y −y −y −y; ϕ*_4_ *= x −x x −x −x x −x x y −y y −y −y y −y y; ϕ*_5_ *= x x x x −x −x −x −x y y y y −y −y −y −y; ϕ*_6_ *= y y y y y y y y −x −x −x −x −x −x −x −x; ϕ*_*r*_ *= x −x −x x −x x x −x −y y y −y y −y −y y*.

The NOE build-up intensities plotted (scaled by the number of protons in the signal) against different mixing times, 10, 50, 150, 250, 400, 550, 700, 850, 1000, 1150, 1300, and 3000 ms, were fitted according to the transient NOE model (Neuhaus, Williamson 1989).^29^ The utilized equation is shown in SI. Furthermore, NOE build-up signals with varying ligand concentrations were used for the determination of *K*_D_ values.

## Results and Discussion

### Ligand Binding Epitope Mapping via ^1^H NOE Pumping

Ligand epitope mapping was achieved from NOE build-up evolution curves from arraying NOE mixing times. As can be seen from the ^1^H NOE pumping NMR spectra in Figure 3, ^1^H signal intensities vary for each ligand, indicating different NOE effects for different proton moieties upon binding with BSA. Glucose, a nonbinding ligand with BSA, was added in each NMR sample solution as an internal control to follow that the observed ligand signals are really originated based on the free translational movement and thus glucose signals should not be observed in ^1^H NOE pumping spectra (Figure 3). Full spectral assignments are provided in SI.

**Figure 3:**
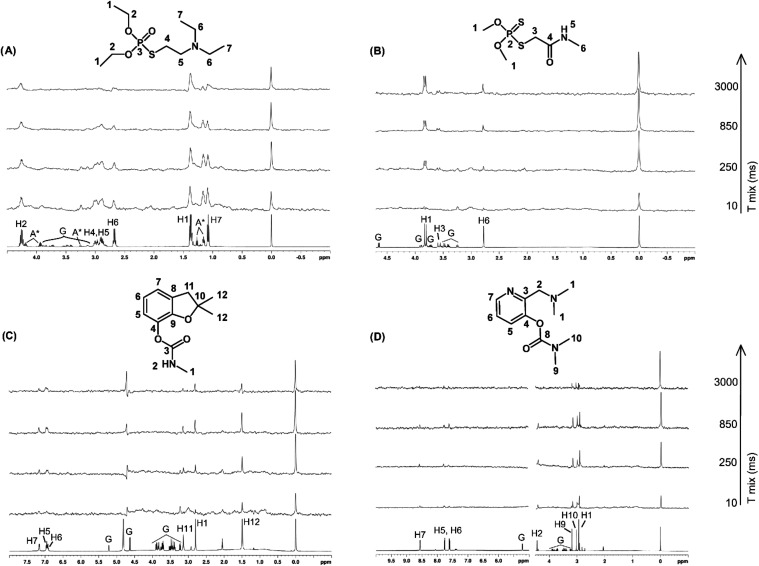
Assigned ^1^H NMR (bottom) and ^1^H NOE pumping NMR spectra with varying mixing times (*t*_mix_ 10–3000 ms) for (A) amiton (amiton synthesis impurities are marked with “A*”), (B) dimethoate, (C) carbofuran, and (D) aminostigmine. Glucose signals are marked with “G.” Glucose was added to the solution as an internal control as it does not have affinity toward albumin, which allows it to be used to control the performance of the NOE pumping experiment via the absence of glucose signals in the ^1^H NOE pumping spectra.

In order to interpret signal intensity changes with respect to increased mixing time, an NOE build-up curve was created for each individual proton signal (Figure 4). For NOE build-up curves, the integrals were scaled according to the number of protons responsible for the particular signal. While the number of ligand protons can be taken into account, the protein side remains unknown. Since the NOE build-up curve is dependent on the spatial proximity between the ligand and the protein upon binding (and also on the number of involved protons within ligand and protein), ligand protons will receive different amounts of NOE upon binding. A stronger NOE signal intensity indicates a closer interhydrogen distance between the ligand and the protein in the bound state. This phenomenon is considered as the basis for mapping ligand epitopes.^30^ To obtain characteristic NOE build-up curves for these ligands, NOE mixing times between 10 and 3000 ms were needed. The fitted build-up curves were utilized to calculate NOE intensities at short mixing time (100 ms), i.e., within the initial rate regime approximation.^31,32^ This approach overcomes the direct signal integration from low signal-to-noise ratio data recorded using short NOE mixing times, allows us to calculate suitable mixing time points, and also uses the whole data series to create the selected time point via curve fitting. In order to utilize initial rate regime analysis via NOE intensities, scaled integrals need to be applied. In our work, NOE signals were calculated at a mixing time of 100 ms, and the signals were normalized against the highest value to give an arbitrary value of 100% to that proton moiety. Thus, ligand protons exhibiting higher percentage values can be considered important for the binding event.

**Figure 4:**
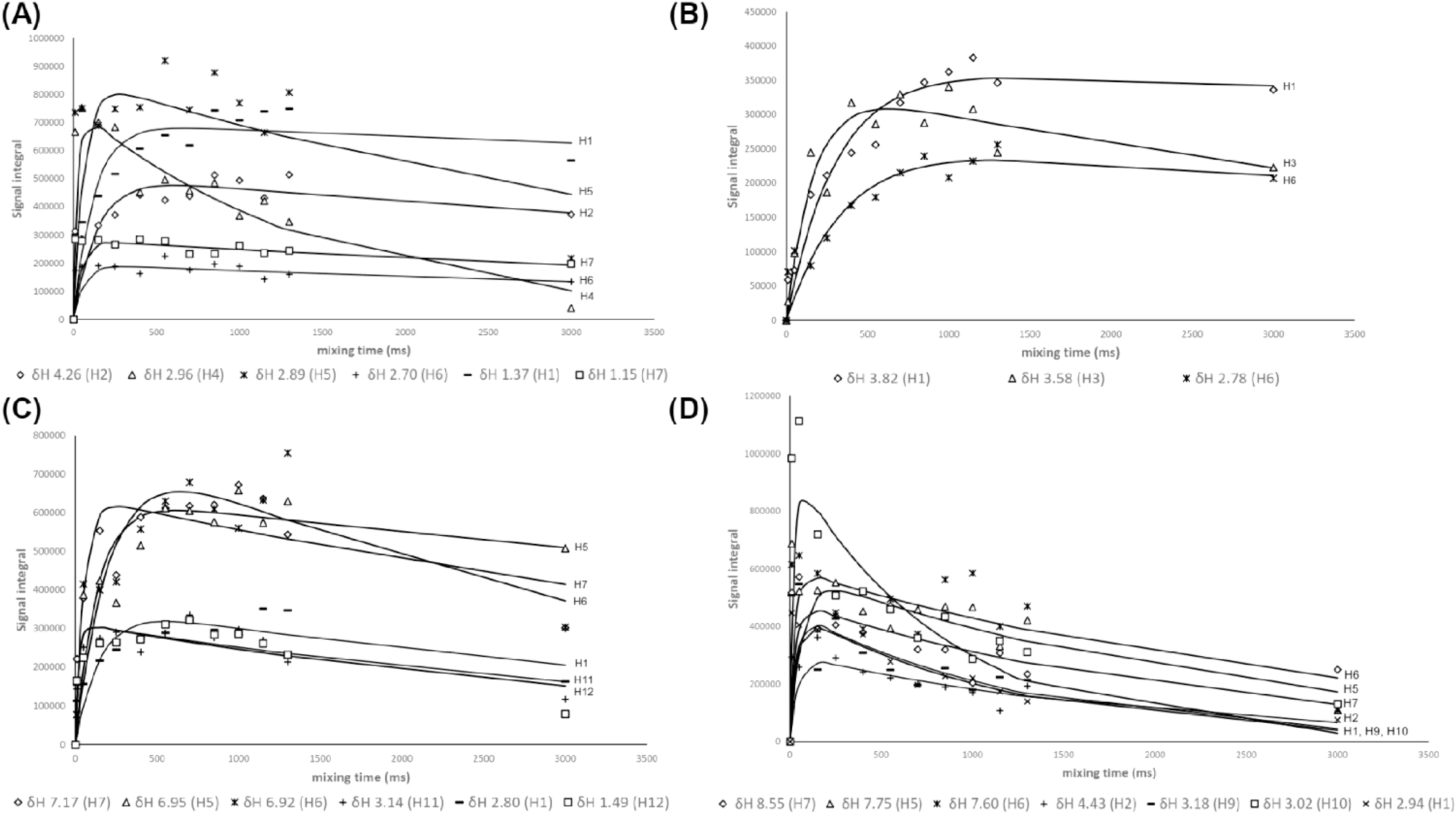
^1^H NOE build-up evolution of (A) amiton (◇ = H2, 4.26 ppm; △ = H4, 2.96 ppm; 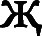 = H5, 2.89 ppm; + = H6, 2.70 ppm; filled rectangle = H1, 1.37 ppm; □ = H7, 1.15 ppm); (B) dimethoate (◇ = H1, 3.82 ppm; △ = H3, 3.58 ppm; 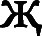 = H6, 2.78 ppm); (C) carbofuran (◇ = H7, 7.17 ppm; △ = H5, 6.95 ppm; 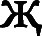 = H6, 6.92 ppm; + = H11, 3.14 ppm; filled rectangle = H1, 2.80 ppm; □ = H12, 1.49 ppm); and (D) aminostigmine (◇ = H7, 8.55 ppm; △ = H5, 7.75 ppm; 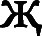 = H6, 7.60 ppm; + = H2, 4.43 ppm; filled rectangle = H9, 3.18 ppm; □ = H10, 3.02 ppm; x = H1, 2.94 ppm); proton signals. Solid lines represent the obtained best fit of the data according to the transient NOE model (Neuhaus, Williamson 1989).^29^ The signal integrals are scaled according to the number of protons within a moiety. Best fit curves were created with Peak-o-mat software.^34^ Sample conditions: ligand (5 mM):BSA (50 μM) ratio 100:1; *T* = 300 K. Amiton (A) and aminostigmine (D) obtain high signal intensities already with short 10 ms NOE mixing times, indicating diffusion filter leakage due to strong ligand interaction with the BSA.

^1^H NOE pumping NMR spectra (Figure 3) display nonzero initial intensities already at the lowest utilized mixing time of 10 ms for amiton (Figure 3A), aminostigmine (Figure 3D), and also for carbofuran (Figure 3C). It seems that ligand signals pass the diffusion filter (optimized for dimethoate), indicating stronger ligand–protein interaction compared with dimethoate. Despite the initial intensities at the lowest mixing time, NOE build-up appears to generally follow the expected intensity behavior with increasing mixing times for carbofuran. Dimethoate behaves optimally throughout the series. Amiton and aminostigmine deviate the most, as some of the protons do not seem to have a build-up region captured in the experiment (masked by initial high intensity), and this leads to ambiguities in NOE build-up curve fitting. Thus, the calculated ligand epitope percentages may not reflect the reliable interpretation for amiton and aminostigmine (Figure 5A,5D). Generally, the highest initial signal intensities for amiton suggest the highest affinity toward BSA of the studied four ligands.

**Figure 5:**
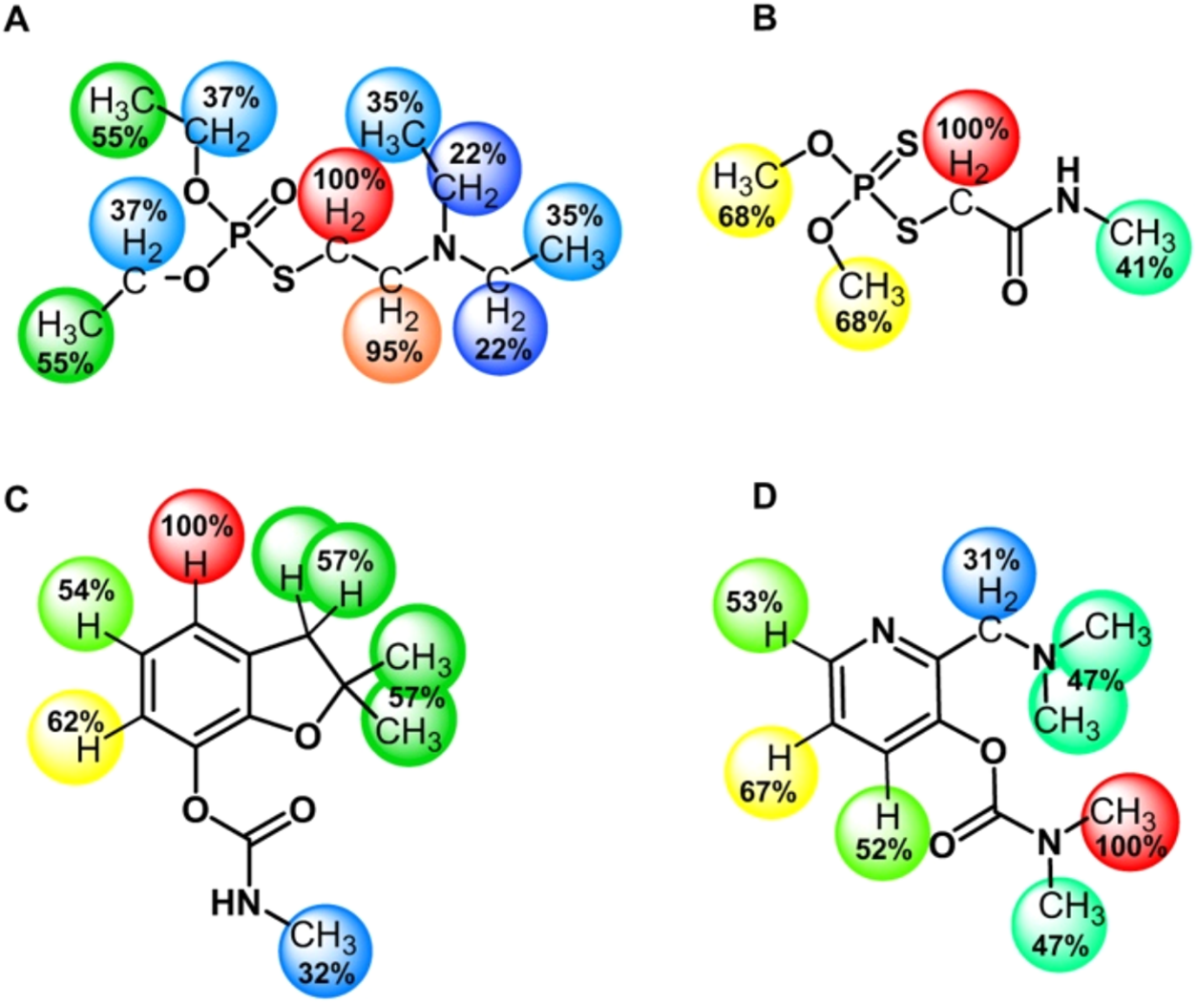
Highlighted binding ligand epitopes with BSA for (A) amiton, (B) dimethoate, (C) carbofuran, and (D) aminostigmine from ^1^H NOE pumping experiments. Percentages and colors (red: high and blue: low) represent relative amounts of intensity from the ^1^H NOE build-up experiments in comparison with a maximum intensity received for the proton moiety within each molecule, which is given a value of 100%. Higher percentage values indicate closer contact with the protein upon binding. Amiton (A) and aminostigmine (D) percentages are likely influenced by the diffusion filter leakage due to strong binding with BSA.

For dimethoate, the highest NOE percentage was obtained for the methylene moiety (H3), indicating a significant role in the binding (Figure 5B). All signals originating from dimethoate also showed the best NOE build-up behavior as the NOE pumping method was originally optimized for this ligand (Figure 4B). For the other OP molecule in this study, amiton, expected NOE intensity behavior was observed only for the H1 and H2 proton moieties in the ethoxy group with NOE percentage observed between 37 and 55%, whereas the calculated NOE percentages for all of the other proton moieties (between 22 and 100%) are possibly influenced by the diffusion filter leakage and the percentage may not reflect reliable interpretation (Figure 5A).

Interestingly, the highest NOE percentage for carbofuran was observed for aromatic proton H7 (Figure 5C). The two other aromatic protons, H5 and H6, also receive substantial NOEs (54–62%), which indicates the closest proximity with the benzene ring upon binding with BSA. Similar to amiton, aminostigmine showed controversial NOE build-up behavior for almost all proton moieties (Figure 4C), originating from diffusion filter leakage due to strong binding, and thus the obtained NOE percentages may not provide reliable information for interpretation (Figure 5D).

### Estimating the *K*_D_ Value from Titration and NOE Pumping Experiments

The capabilities of the ^1^H NOE pumping experiment were tested to determine dissociation constant values *K*_D_ for the presented molecules A–D (Figure 1) as a proof of concept. The dissociation constant *K*_D_ (*K*_D_ = 1/*K*_A_, association constant *K*_A_) for each molecule was evaluated by combined titration and ^1^H NOE pumping experiments (Figures S23–S26). A global *K*_D_ value for each compound was calculated using the scaled integral data of all signals.^35^ A fit of the 1:1 binding model (described in SI) was utilized to estimate global *K*_D_ values for each ligand with BSA (Table S3). As no repetitive measurements were performed, the obtained *K*_D_ values are thus considered preliminary. All studied ligands showed *K*_D_ values within a 1–7 mM range (amiton 3.0 mM, dimethoate 5.1 mM, carbofuran 1.6 mM, and aminostigmine 6.6 mM), although the obtained *K*_D_ values regarding those ligands that show strong binding in the NOE pumping experiment (high intensities at the lowest mixing times) need to be interpreted warily, i.e., amiton and aminostigmine. These ligands would be expected to show much lower *K*_D_ values than dimethoate and carbofuran, both of which were showing normally behaving NOE pumping data. It remains speculative whether the obtained *K*_D_ values for amiton and aminostigmine represent some secondary binding to BSA. This observation underscores a significant concern regarding the utilization of *K*_D_ values obtained using the NOE pumping method and emphasizes the necessity for further investigation, especially concerning the binding sites and the ramifications of such interactions on drug transport, an essential function of both BSA and HSA. Moreover, the findings align with molecular modeling results, indicating that binding interactions with BSA are indeed occurring.^36^ This data also prompts an inquiry into the implications of carbamate compounds themselves and their potential hazards as insecticides as well. There is published data regarding the binding affinity of OP-protein, which is reported in a range of 10^–3^ to 10^–5^ M.^37^ The results for amiton and dimethoate are in agreement with those previously reported, whereas the *K*_D_ values for carbofuran and aminostigmine obtained by NMR spectroscopy are explored for the first time.

### Ligand Competition Binding with BSA from ^1^H NOE Pumping

A study by Salvia et al.^38^ has shown that the typically employed techniques in NMR mixture analysis include STD NMR, diffusion-ordered spectroscopy (DOSY), and total correlation spectroscopy (TOCSY). To highlight the full potential of the ^1^H NOE pumping method, we applied the method in the NMR mixture analysis of these toxic ligands. In the NMR chemosensing study by Salvia et al., the ^1^H NOE pumping experiment was the only applied method, which provided unambiguous identification of an analyte interacting selectively with the nanoparticles in a complex mixture. In our study, the ligand mixture analysis with BSA was carried out using the ^1^H NOE pumping method for the dimethoate/carbofuran mixture (and glucose control) (Figures S27 and S28). Our data show that both ligands can be identified from the ^1^H NOE pumping spectrum, and we were able to obtain NOE build-up curves for carbofuran and dimethoate (Figure S28) in the mixture. This would enable a data comparison between individual and ligand mixture experiments. In general, it is known that if one ligand shows a higher binding affinity against the binding sites of the target protein, the receptive signals for the other ligands will either decrease or increase.^39^ This encouraged us to investigate whether these ligands were competing for the same binding sites on BSA, particularly given the inclusion of two different types of ligands (OP and CM) without conducting any site marker experiments with the BSA binding sites in three different subdomains (I–IIIA and B). By inspection of the NMR spectra (Figure S28) from the two-component system with dimethoate (OP) and carbofuran (CM), the obtained NOE build-up curves and especially the receptive signal intensities drastically differ, where signals from dimethoate decrease, indicating that these two ligands might be competing for the same BSA binding site. According to the literature, ligands may bind to BSA sites, which are located in the hydrophobic cavities found in BSA subdomains IIA and IIIA.^40^ Moreover, many OP pesticides, e.g., parathion and diazinon, are known to prefer binding to BSA binding site I located in the BSA subdomain IIA.^10^ Our data would hence indicate that the binding for carbofuran also takes place on BSA site I in subdomain IIA. The obtained results are also in agreement with previously reported findings.^36^ The outcome emphasizes the ability of the ^1^H NOE pumping experiment to be applied in complex mixture analysis, especially with glucose as a nonbinding internal control ligand, thereby minimizing the likelihood of false-positive results.

## Conclusions

In this work, atomic-level information regarding the binding of four toxic chemicals, amiton, dimethoate, carbofuran, and aminostigmine, with BSA was revealed using ^1^H NOE pumping NMR experiments. Preliminary ligand epitope mapping analysis was performed for the first time, as well as the determination of the *K*_D_ values for the studied compounds solely utilizing NMR spectroscopy. Although the observed *K*_D_ values showed binding strength within the range of 1–7 mM, the characteristic NOE signal behavior for amiton and aminostigmine in the NOE pumping experiment revealed stronger binding. In addition, the competitive binding dynamics between dimethoate and carbofuran for a shared binding site on BSA was illustrated, thereby underscoring the efficacy of the ^1^H NOE pumping method in general. Our initial experimental results herein necessitate further studies, particularly involving repetitive and optimized *K*_D_ studies and a broader ligand portfolio between OP and CM compounds. This research contribution enhances our understanding of the binding interactions of these compounds with serum proteins and underscores the relevance of employing advanced spectroscopic techniques in pharmacological research.

## Supplementary Material



## Notes

Additional figures for characterization (NMR spectra), table of complete chemical shift assignments for the tested compounds, the dissociation constant results, ^1^H NOE pumping NMR spectra, NOE build-up curves for ligand competition binding, utilized equations as well as ^1^H NOE pumping pulse sequence (PDF)
