# Supplementary material for: Revealing Organophosphorus and Carbamate Interactions with Albumin Using 1H NOE Pumping NMR Technique
Source: Anal Chem. 2026 Jan 28;98(5):3493–500. doi: 10.1021/acs.analchem.5c02132 (PMC12903051; doi:10.1021/acs.analchem.5c02132)
Supplement: Supplementary file 1 [file ac5c02132_si_001.pdf]

Revealing organophosphorus and carbamate interactions with albumin using ^{1}H NOE pumping NMR technique

Ivana Sofrenić[1,2], Sami Heikkinen[3*], Anne Puustinen[1], Niko Minkkinen[1], Harri Kiljunen[1] and Harri A. Heikkinen[1*]

[1] VERIFIN, University of Helsinki, P.O. Box 65, FIN-00014 Helsinki, Finland. * Corresponding author.
[2] University of Belgrade, Faculty of Chemistry, Studentski trg 12-16, 11000 Belgrade, Serbia.
[3] Department of Chemistry, University of Helsinki, P.O. Box 65, FIN-00014 Helsinki, Finland.

ABSTRACT

In this work, the capability of ^{1}H Nuclear Overhauser Effect (NOE) pumping NMR technique was applied to elucidate the atomic level binding interaction between the bovine serum albumin (BSA) and four toxic compounds: amiton, dimethoate, carbofuran and aminostigmine. With the aid of ^{1}H NOE pumping experiments, we were able to highlight ligand binding epitopes for the studied compounds and provide prefatory data for the ligand affinity with BSA via the dissociation constant values (K_D). In addition, we demonstrate that the ^{1}H NOE pumping technique is suitable for the ligand competition studies solely using one NMR sample, and that the technique is a simple and straightforward method capable of revealing important parameters that are used typically to define ligand – albumin interaction at the atomic level. We believe, the novel precursory results herein provide important and experimentally driven data for the BSA interaction especially for carbamate-based molecules, where the existing literature is fairly limited. Based on the preliminary experimental results, amiton and aminostigmine showed stronger binding to BSA based on NOE pumping data compared to dimethoate and carbofuran although the obtained K_D values were observed within the similar range. Our results present the first comparable study between the organophosphorus (OP) and the carbamate (CM) toxic compounds with BSA via NMR spectroscopy only. Furthermore, the efficacy of the ^{1}H NOE pumping technique provided evidence that the organophosphorus and carbamate compounds bind to a common epitope site on BSA.

*Corresponding Authors

Harri A. Heikkinen

VERIFIN, Department of Chemistry, University of Helsinki, P.O. Box 65, FIN-00014 Helsinki, Finland.

Sami Heikkinen

Department of Chemistry, University of Helsinki, P.O. Box 65, FIN-00014 Helsinki, Finland.

List of contents

Figure S1. The ^{1}H NMR spectrum of amiton in D_2O with assignments.4

Figure S2. The ^{31}P NMR spectrum of amiton in D_2O.4

Figure S3. The 2D [^{1}H-^{1}H]-COSY NMR spectrum of amiton in D_2O.5

Figure S4. The 2D [^{1}H-^{1}H]-NOESY NMR spectrum of amiton in D_2O.5

Figure S5. The 2D [^{1}H-^{13}C]-HSQC NMR spectrum of amiton in D_2O.6

Figure S6. The 2D [^{1}H-^{13}C]-HMBC NMR spectrum of amiton in D_2O.6

Figure S7. The 2D [^{1}H-^{31}P]-HMQC NMR spectrum of amiton sample in D_2O with assignments.7

Figure S8. The ^{1}H NMR spectrum of dimethoate in D_2O with assignments.8

Figure S9. The ^{31}P NMR spectrum of dimethoate in D_2O.8

Figure S10. The 2D [^{1}H-^{13}C]-HSQC NMR spectrum of dimethoate in D_2O.9

Figure S11. The 2D [^{1}H-^{13}C]-HMBC spectrum of dimethoate in D_2O.9

Figure S12. The 2D [^{1}H-^{31}P]-HMQC spectrum of dimethoate in D_2O.10

Figure S14. The 2D [^{1}H-^{1}H]-COSY NMR spectrum of carbofuran in CD_3CN.11

Figure S15. The 2D [^{1}H-^{13}C]-HSQC NMR spectrum of carbofuran in CD_3CN.11

Figure S16. The 2D [^{1}H-^{13}C]-HMBC NMR spectrum of carbofuran in CD_3CN.12

Figure S17. The ^{1}H NMR spectrum of aminostigmine in CD_3CN with assignments.12

Figure S18. The ^{13}C NMR spectrum of aminostigmine in CD_3CN.13

Figure S19. The 2D [^{1}H-^{1}H]-COSY NMR spectrum of aminostigmine in CD_3CN.13

Figure S20. The 2D [^{1}H-^{1}H]-NOESY NMR spectrum of aminostigmine in CD_3CN.14

Figure S21. The 2D [^{1}H-^{13}C]-HSQC NMR spectrum of aminostigmine in CD_3CN.14

Figure S22. The 2D [^{1}H-^{13}C]-HMBC NMR spectrum of aminostigmine in CD_3CN.15

Table S1. The ^{1}H, ^{13}C and ^{31}P NMR data for tested compounds.16

Table S2. The ^{1}H, ^{13}C and ^{31}P NMR data of synthesis impurities in the amiton sample.17

Figure S23. The ^{1}H NOE build-up evolution curves of amiton18

Figure S24. The ^{1}H NOE build-up evolution curves of dimethoate19

Figure S25. The ^{1}H NOE build-up evolution curves of carbofuran20

Figure S26. The ^{1}H NOE build-up evolution curves of aminostigmine21

Table S3. The obtained dissociation constants K_D for OP- and CM-BSA interactions.21

Figure S27. The ^{1}H NMR spectra with assignation of protons for carbofuran and dimethoate and ^{1}H NOE pumping NMR spectra.22

Figure S28. The NOE build-up evolution of carbofuran and dimethoate**Error! Bookmark not defined.**

Pulse sequence.24
Equations29

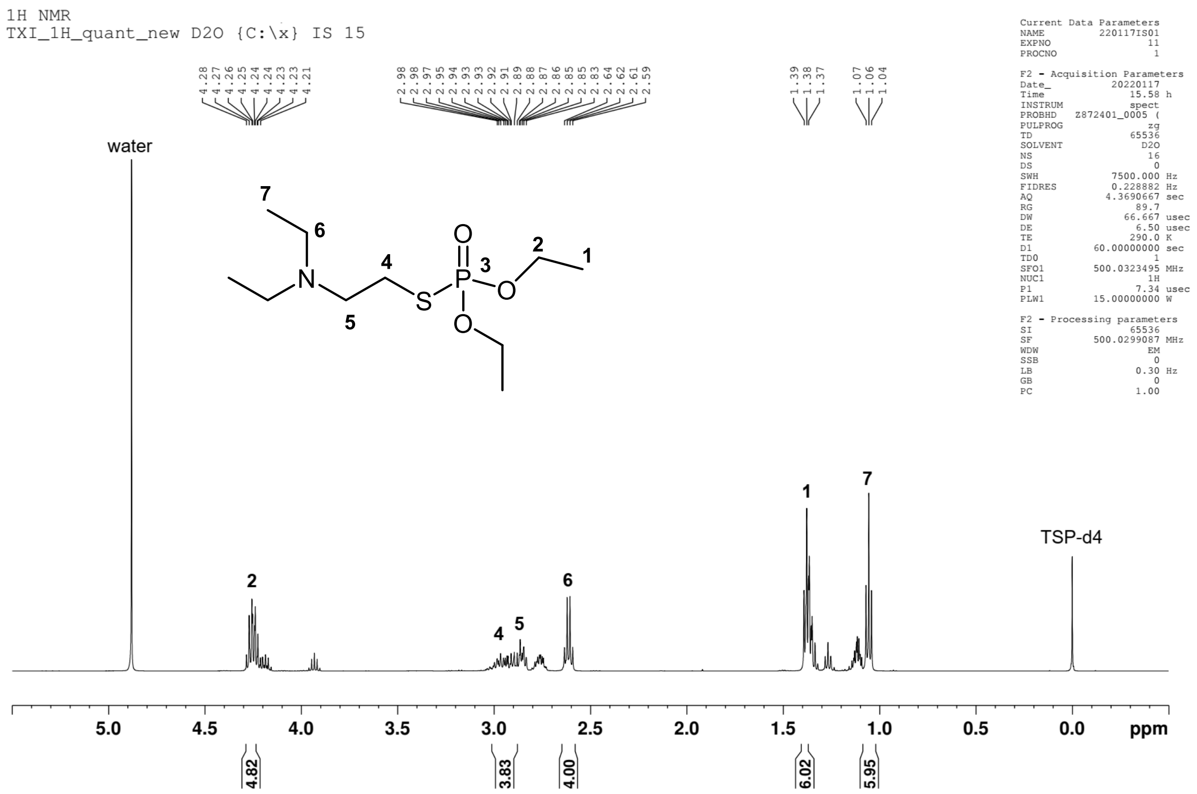

Figure S1. The ^{1}H NMR spectrum of amiton in D_2O with assignments.

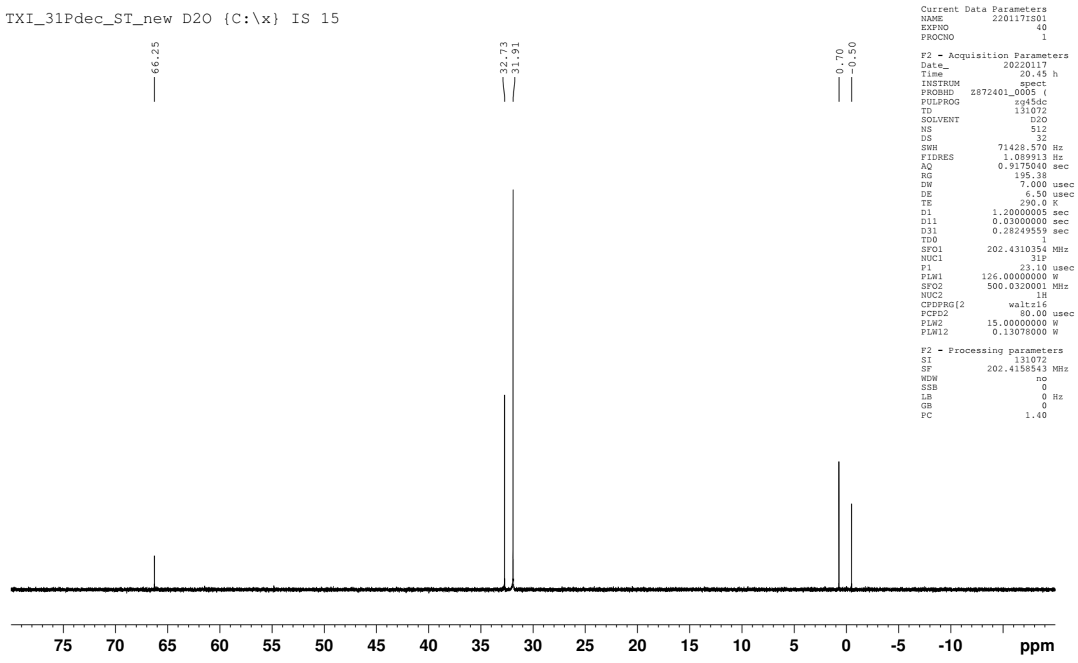

Figure S2. The ^{31}P NMR spectrum of amiton in D_2O.

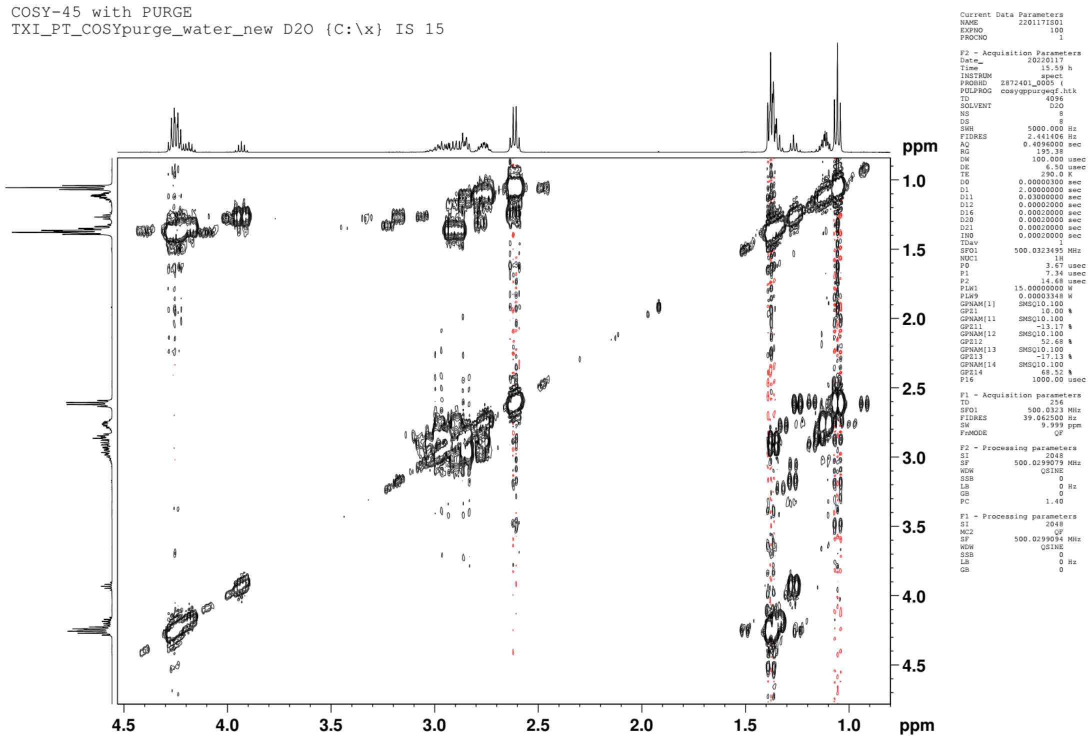

Figure S3. The 2D [^{1}H-^{1}H]-COSY NMR spectrum of amiton in D_2O.

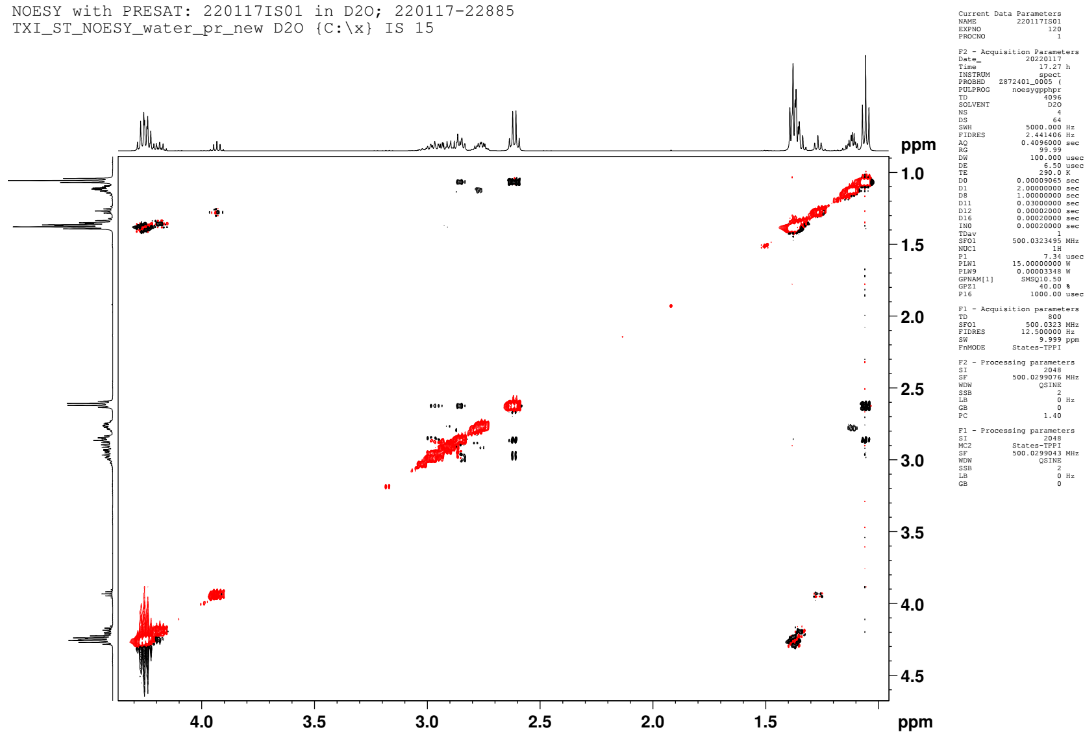

Figure S4. The 2D [^{1}H-^{1}H]-NOESY NMR spectrum of amiton in D_2O.

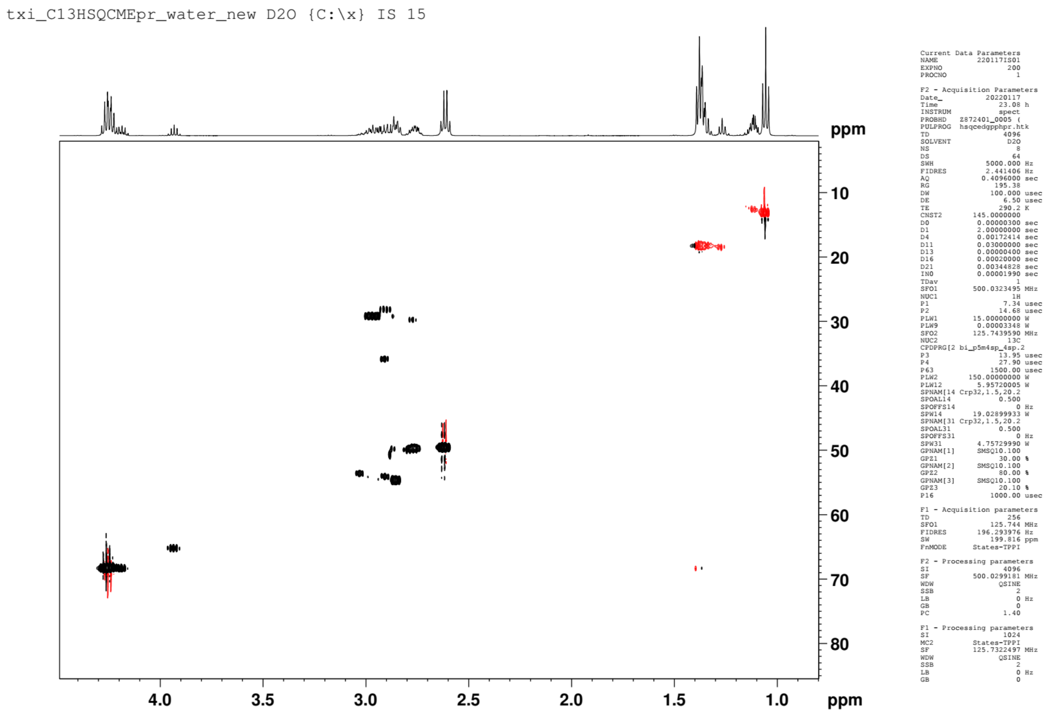

Figure S5. The 2D [^{1}H-^{13}C]-HSQC NMR spectrum of amiton in D_2O.

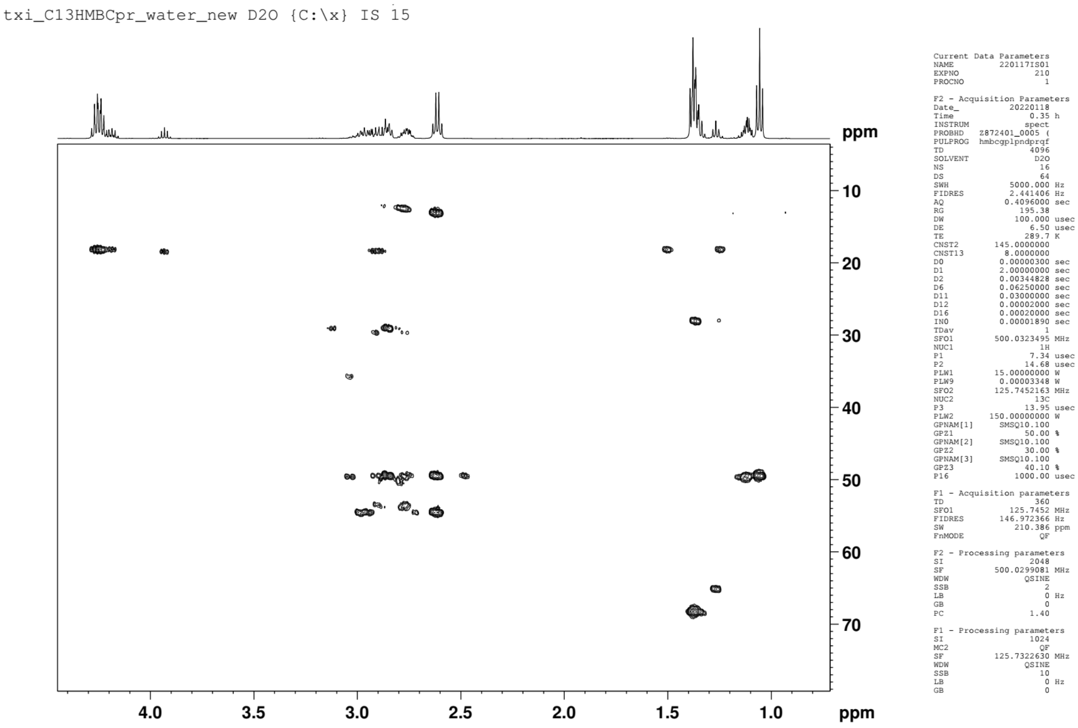

Figure S6. The 2D [^{1}H-^{13}C]-HMBC NMR spectrum of amiton in D_2O.

TXI_2DHP_PT_new2 D2O {C:\x} IS 15

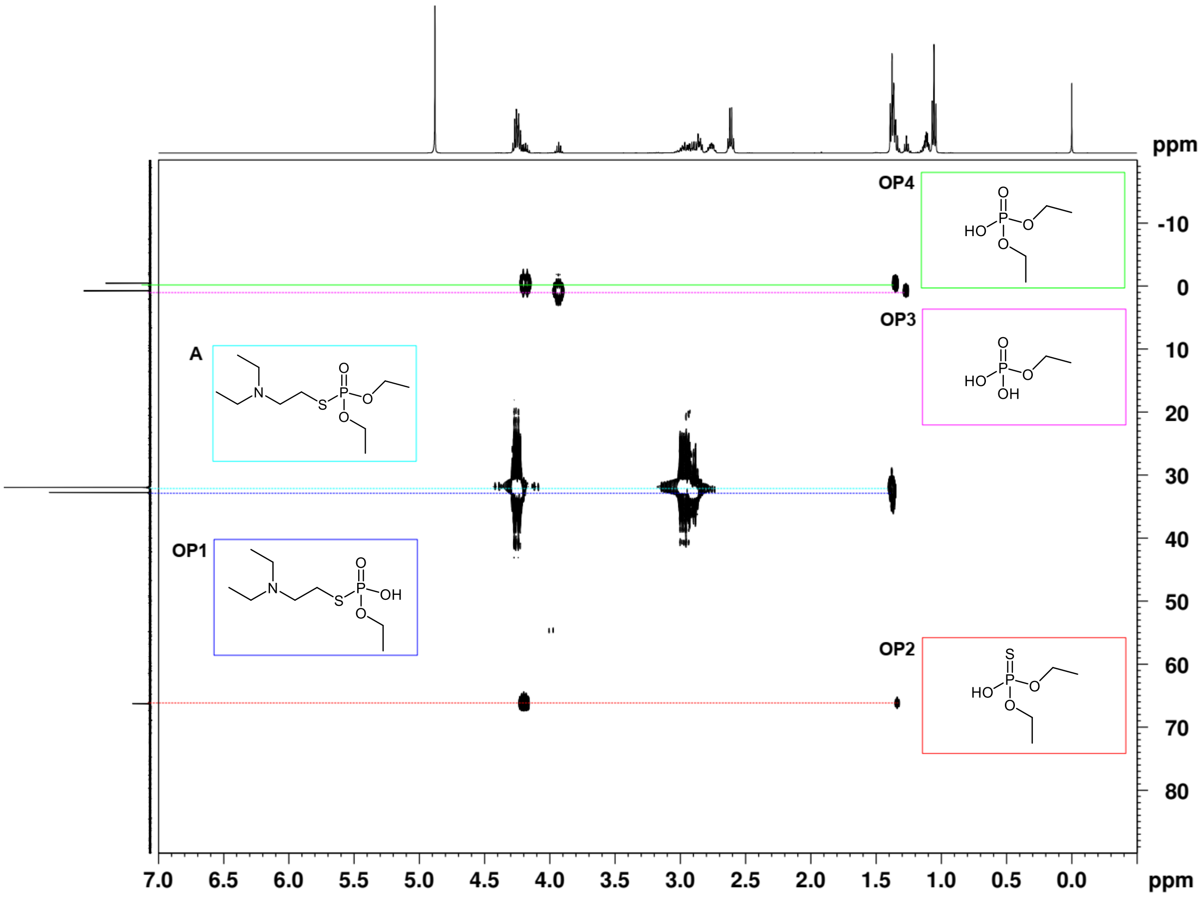

Figure S7. The 2D [^{1}H-^{31}P]-HMQC NMR spectrum of amiton sample in D_2O with assignments.

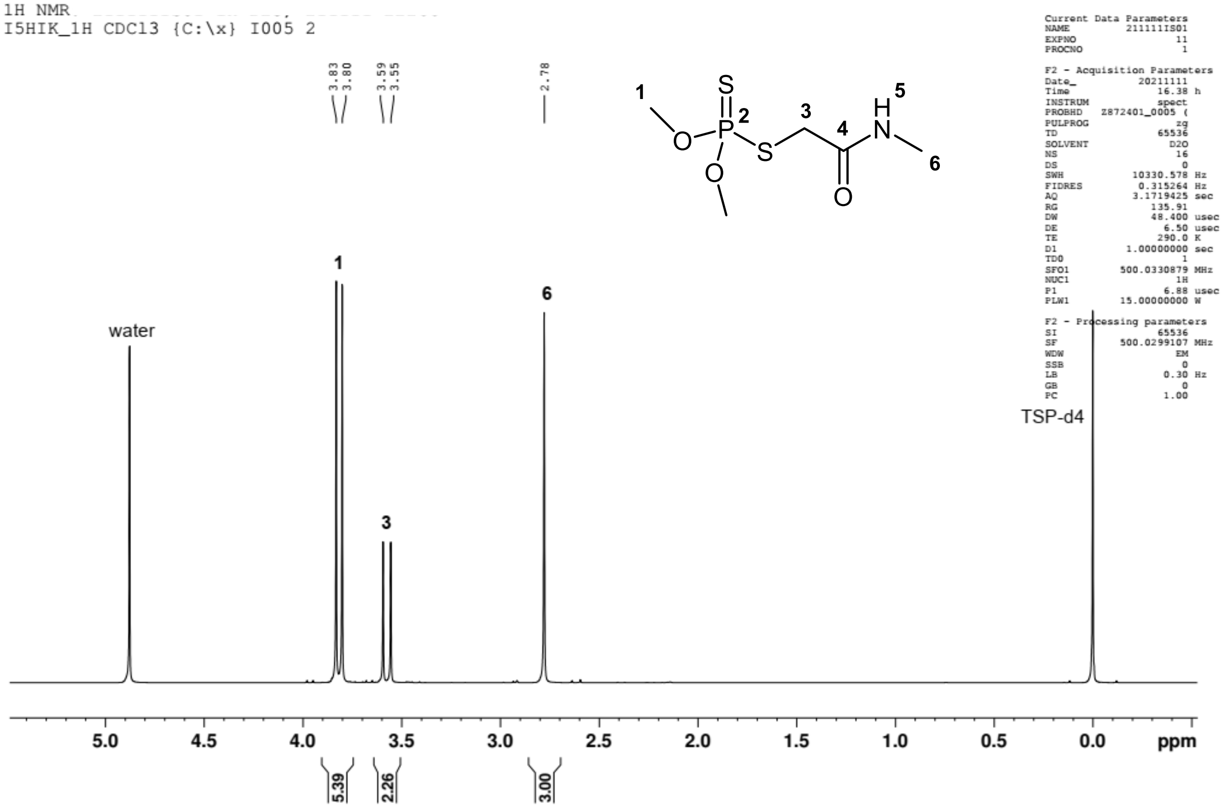

Figure S8. The ^{1}H NMR spectrum of dimethoate in D_2O with assignments.

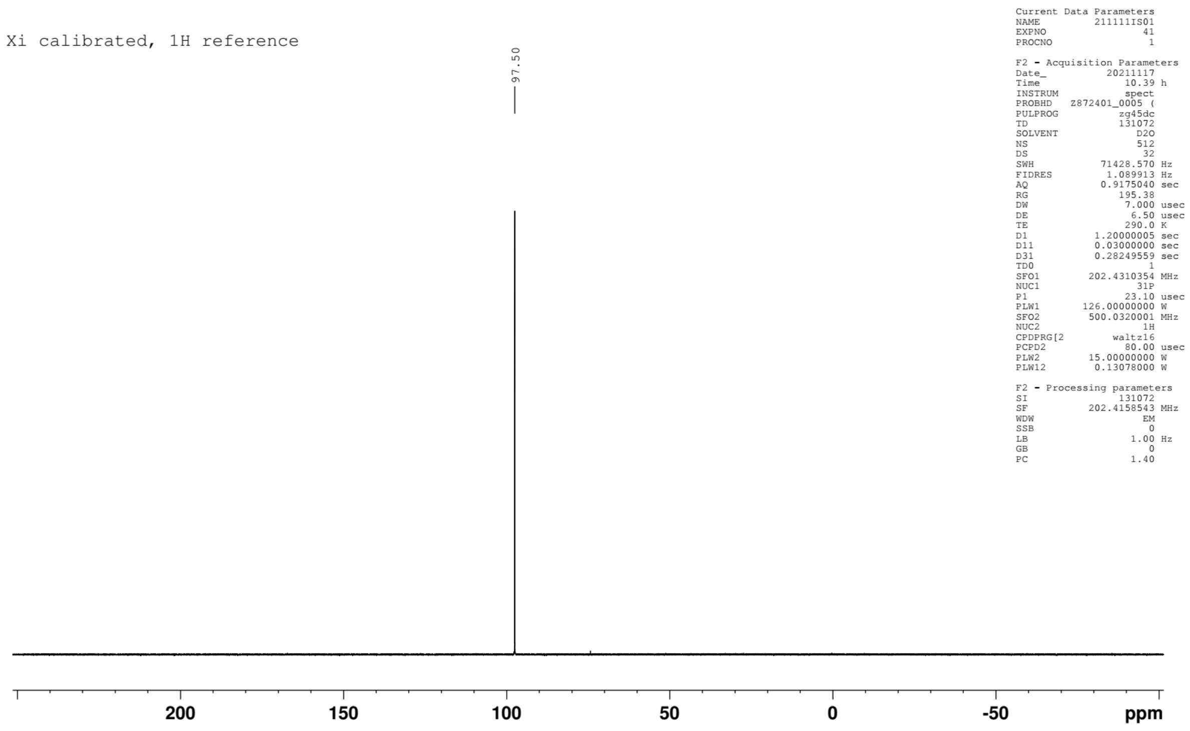

Figure S9. The ^{31}P NMR spectrum of dimethoate in D_2O.

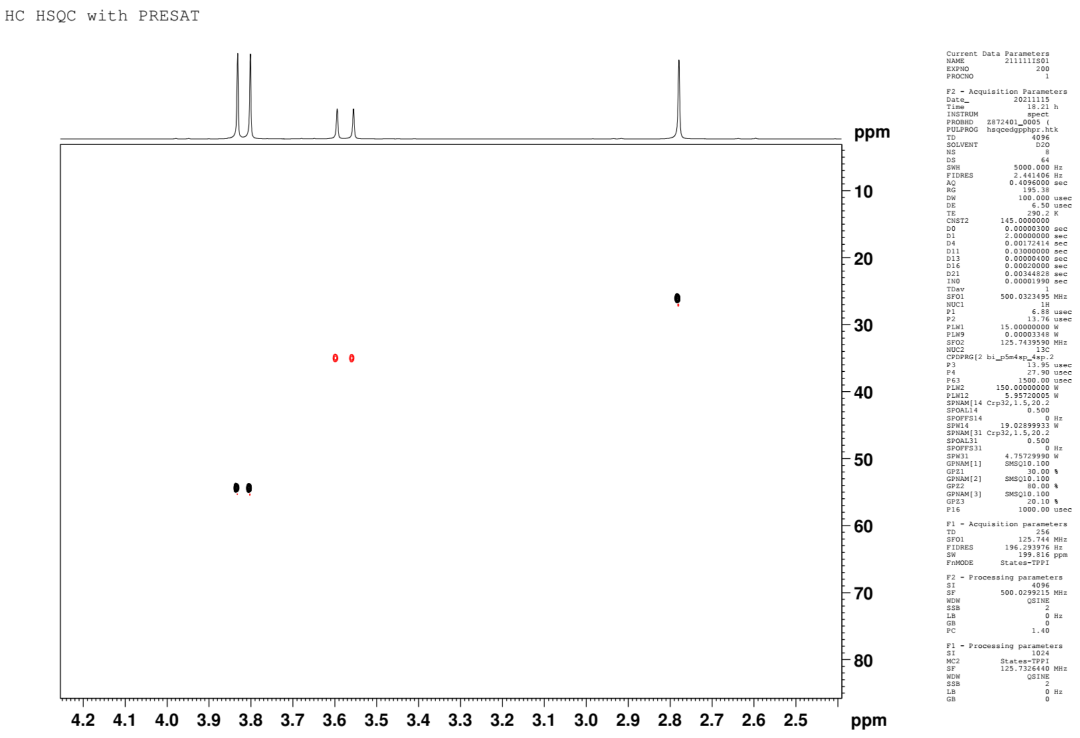

Figure S10. The 2D [^{1}H-^{13}C]-HSQC NMR spectrum of dimethoate in D_2O.

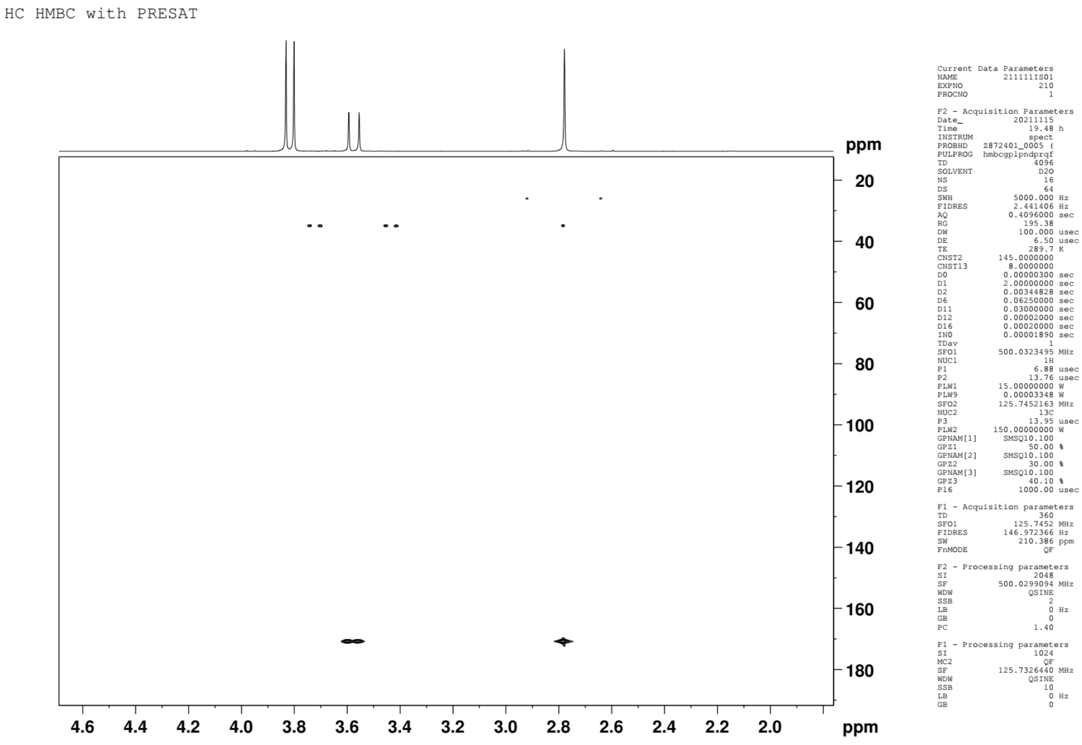

Figure S11. The 2D [^{1}H-^{13}C]-HMBC spectrum of dimethoate in D_2O.

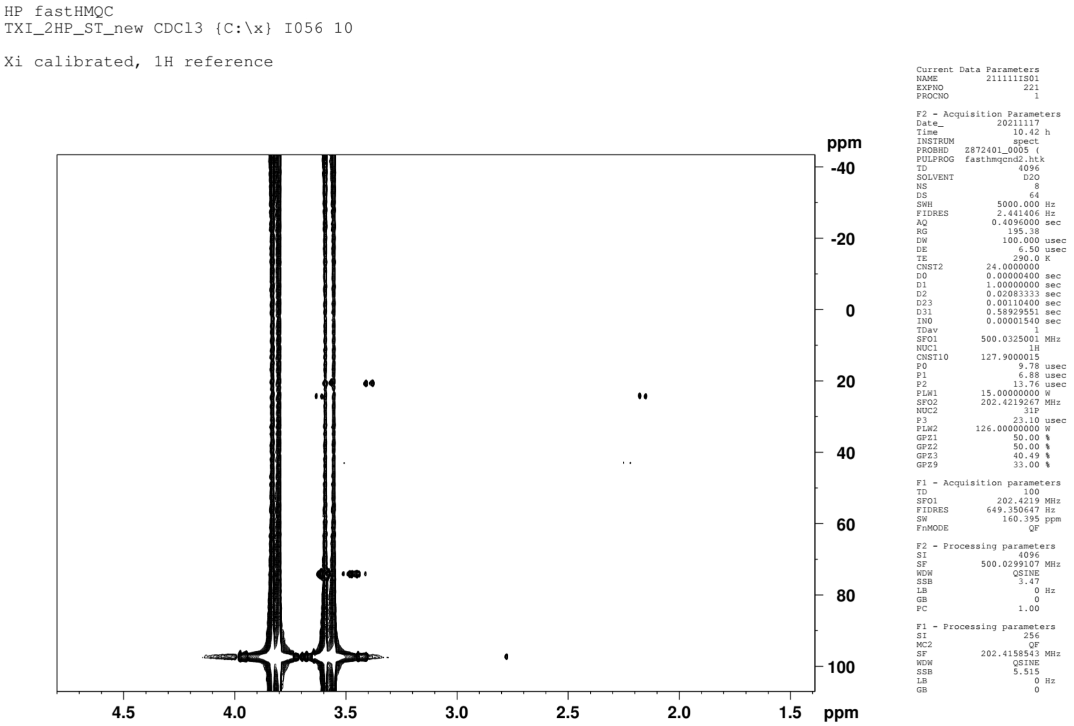

Figure S12. The 2D [^{1}H-^{31}P]-HMQC spectrum of dimethoate in D_2O.

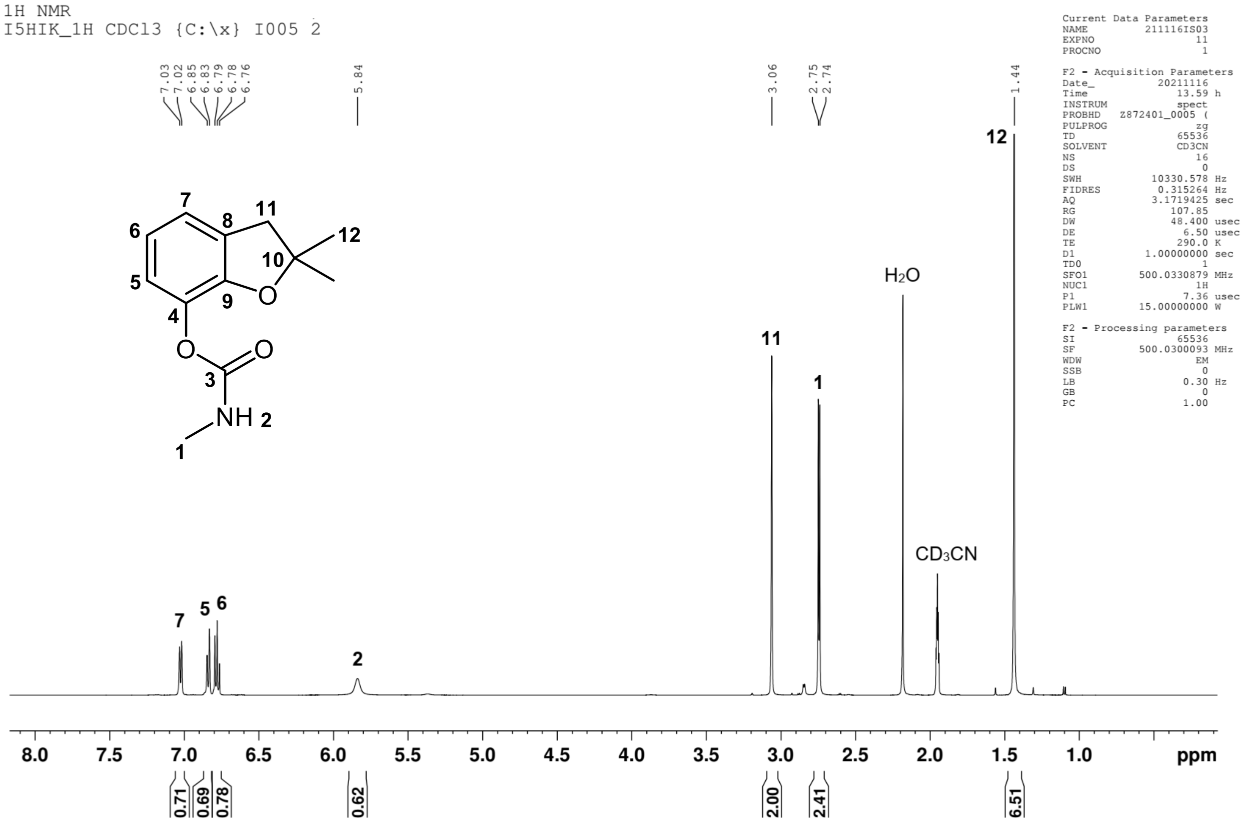

Figure S13. The ^{1}H NMR spectrum of carbofuran in CD_3CN with assignment.

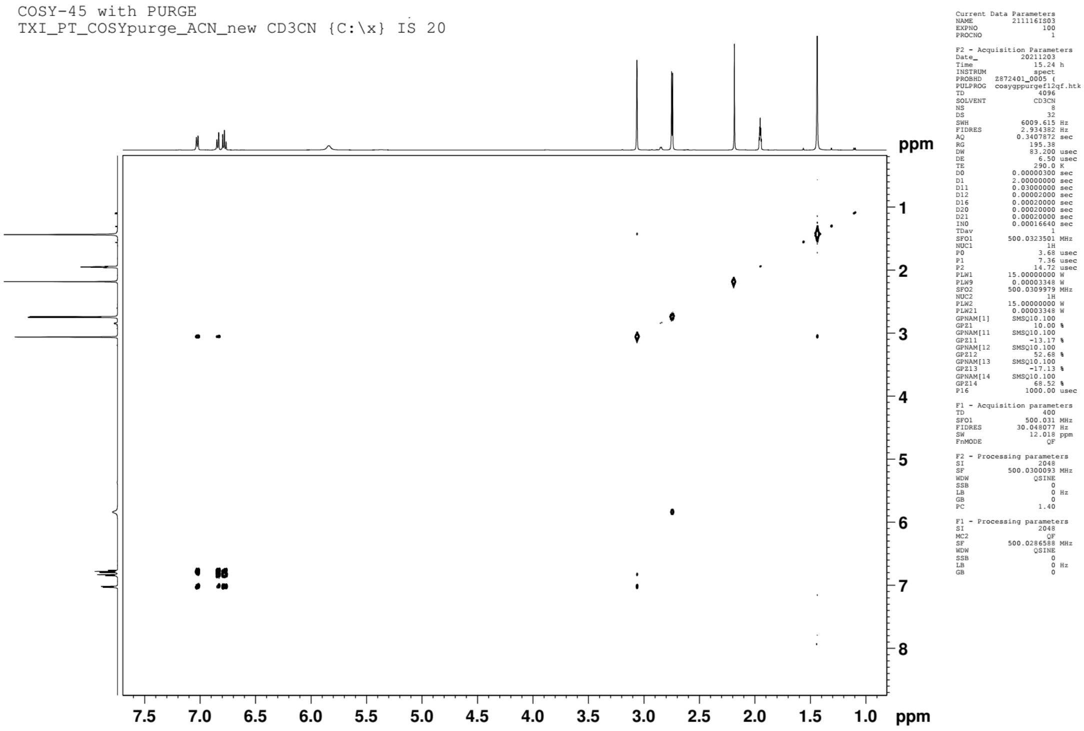

Figure S14. The 2D [^{1}H-^{1}H]-COSY NMR spectrum of carbofuran in CD_3CN.

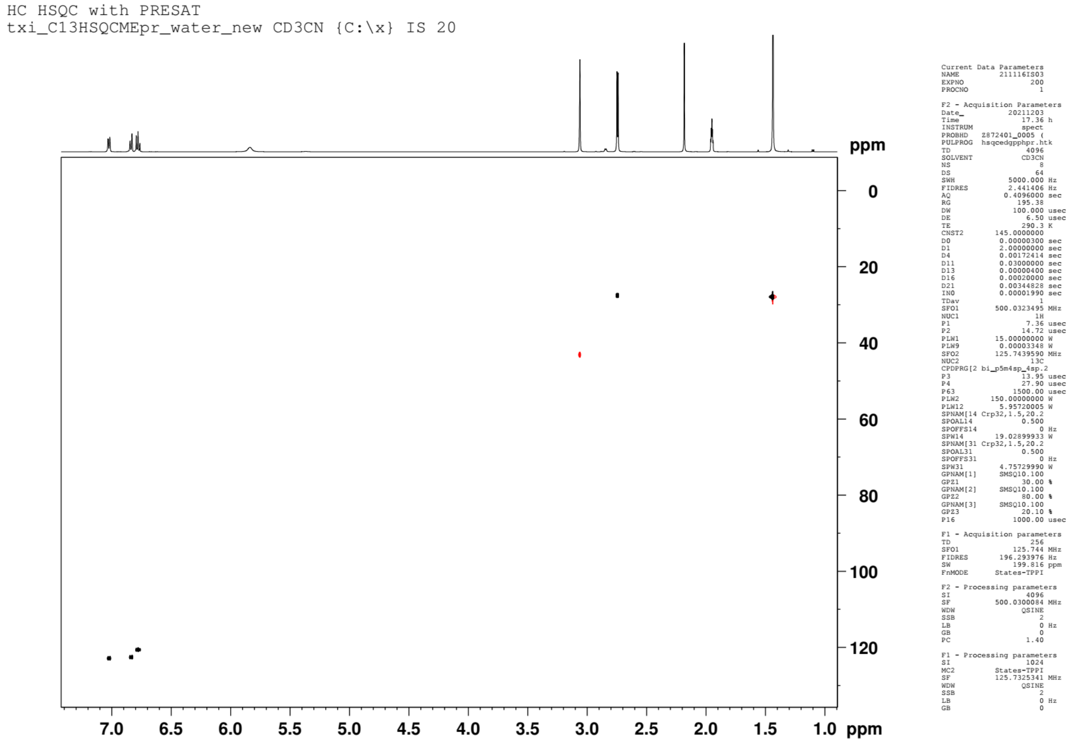

Figure S15. The 2D [^{1}H-^{13}C]-HSQC NMR spectrum of carbofuran in CD_3CN.

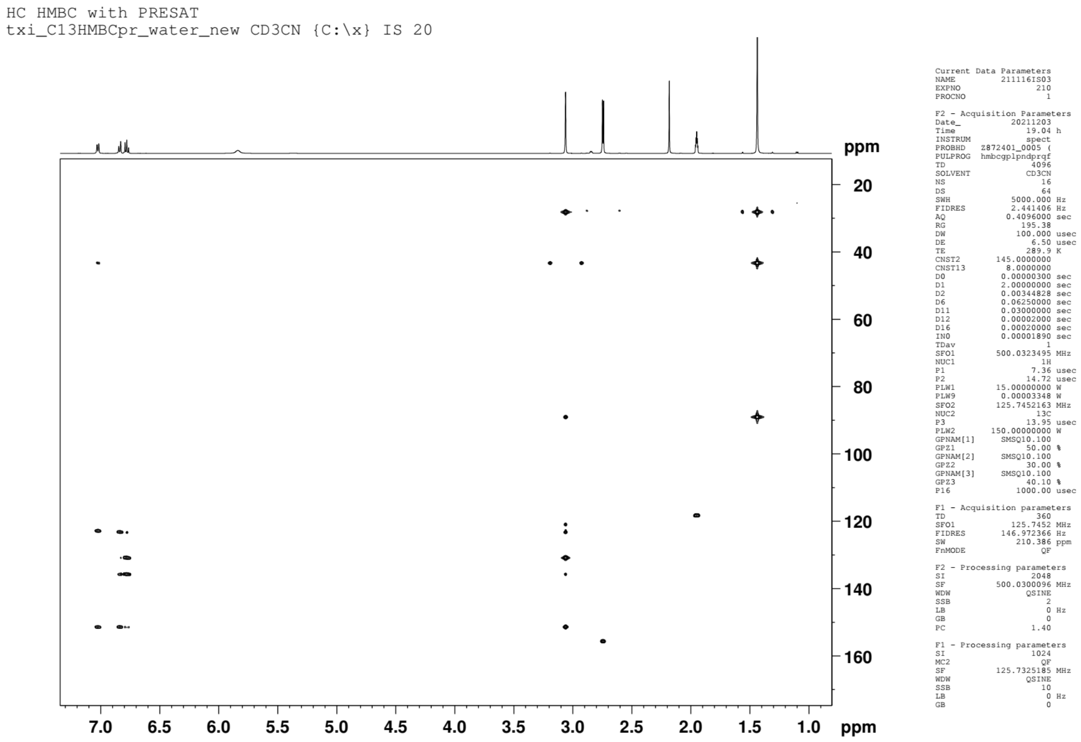

Figure S16. The 2D [^{1}H-^{13}C]-HMBC NMR spectrum of carbofuran in CD_3CN.

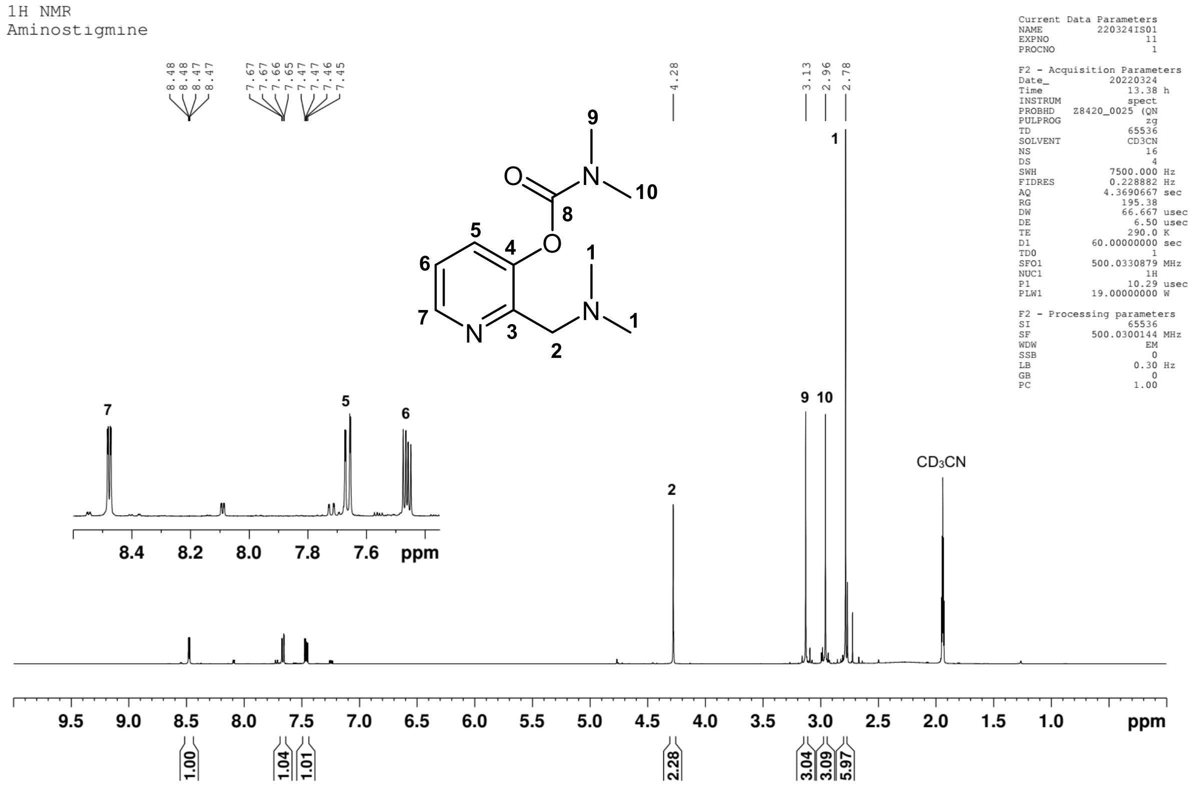

Figure S17. The ^{1}H NMR spectrum of aminostigmine in CD_3CN with assignments.

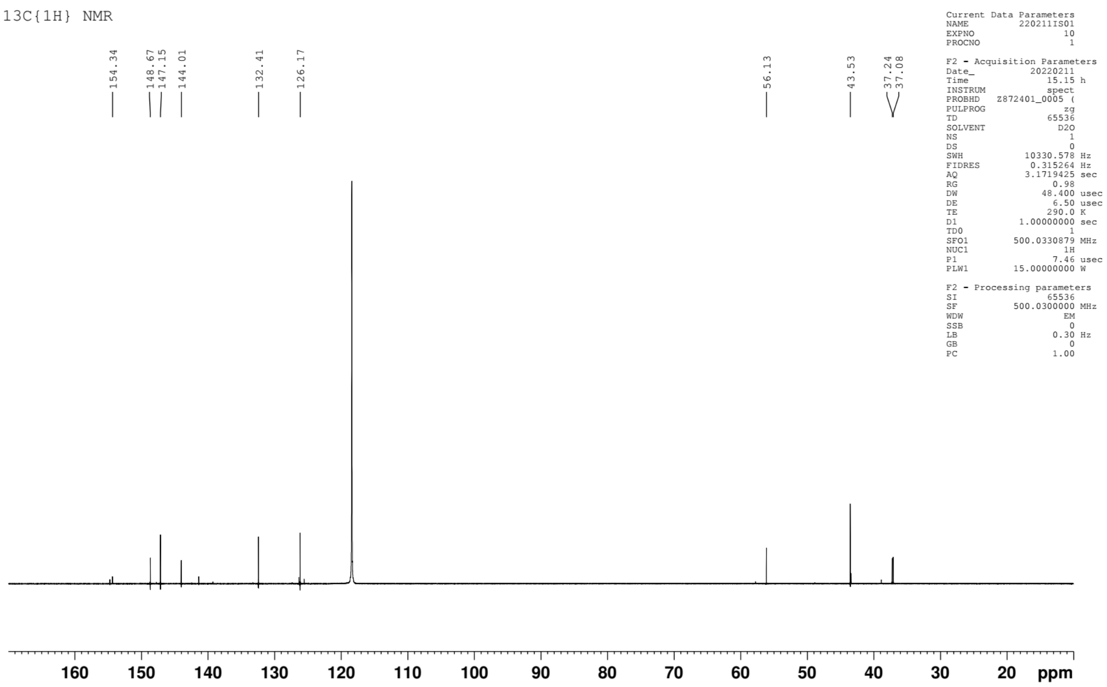

Figure S18. The ^{13}C NMR spectrum of aminostigmine in CD_3CN.

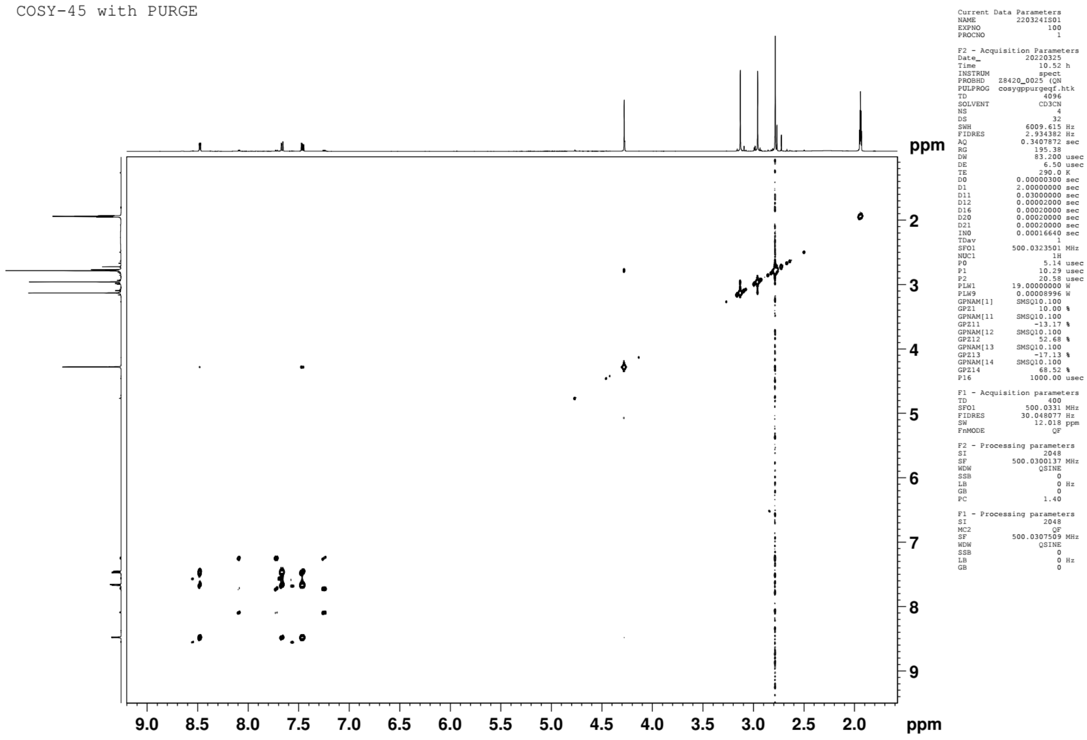

Figure S19. The 2D [^{1}H-^{1}H]-COSY NMR spectrum of aminostigmine in CD_3CN.

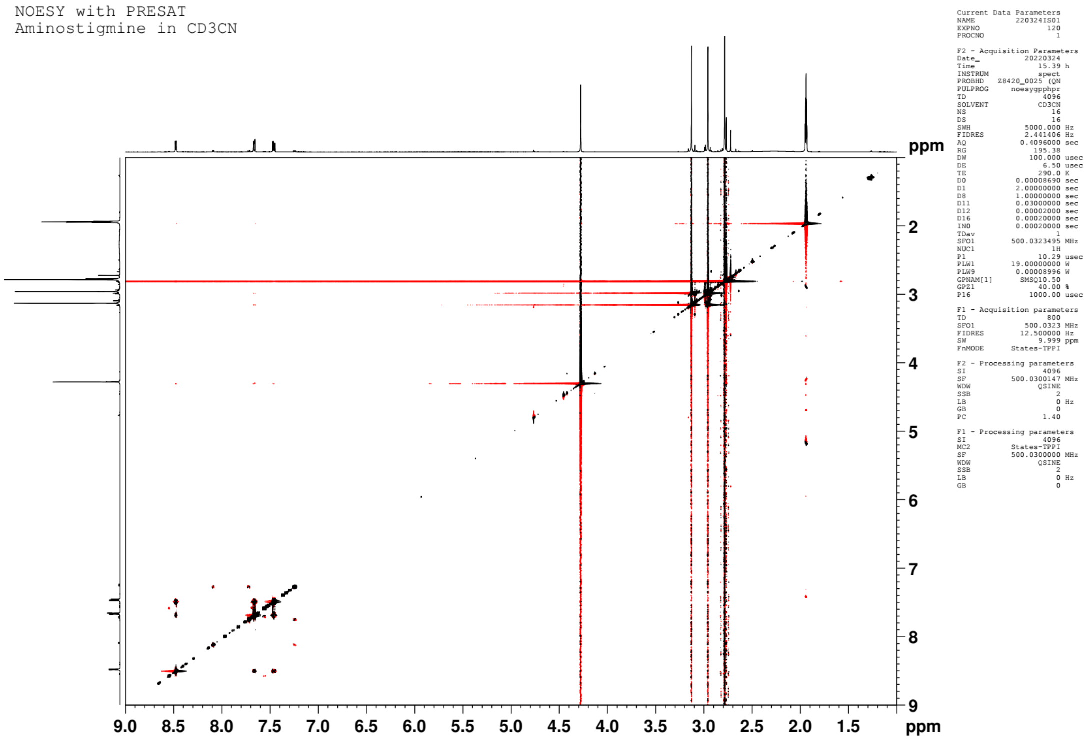

Figure S20. The 2D [^{1}H-^{1}H]-NOESY NMR spectrum of aminostigmine in CD_3CN.

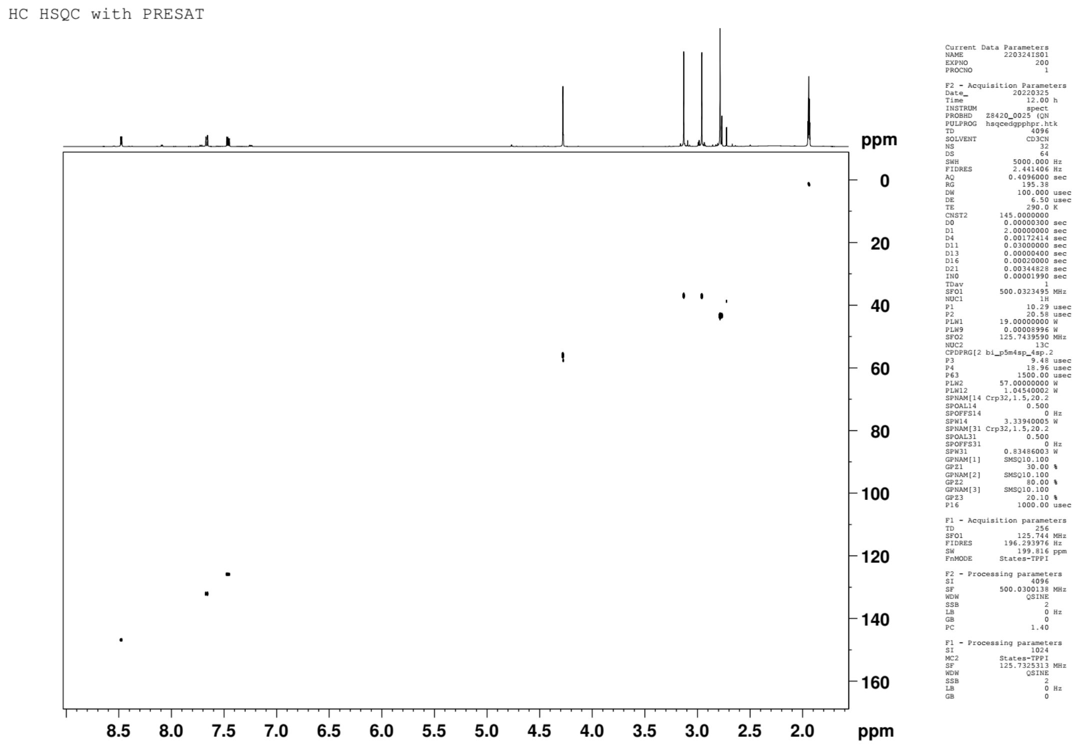

Figure S21. The 2D [^{1}H-^{13}C]-HSQC NMR spectrum of aminostigmine in CD_3CN.

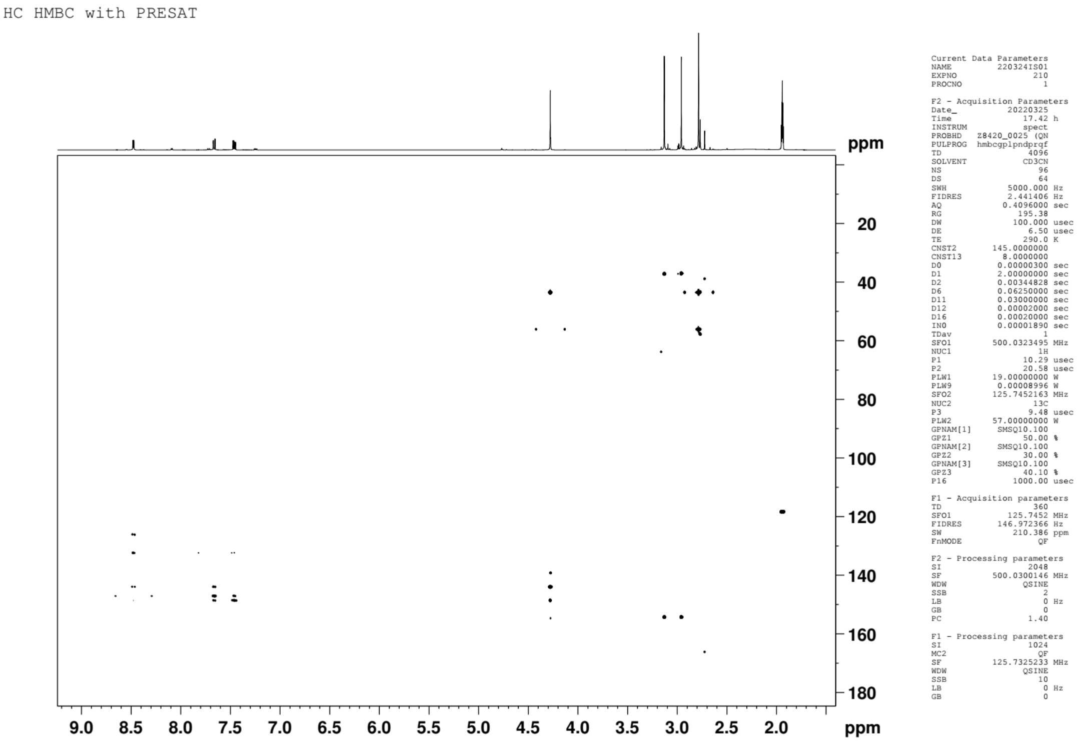

Figure S22. The 2D [^{1}H-^{13}C]-HMBC NMR spectrum of aminostigmine in CD_3CN.

Table S1. The ^{1}H, ^{13}C and ^{31}P NMR data for tested compounds.

Compound	No.	δ_C, ppm	δ_H, ppm (J, Hz)	δ_P, ppm
**Amiton**				
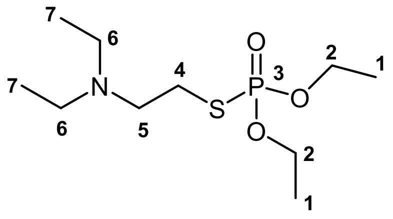	1	18.1	1.38, t (6.9)	
	2	68.4	4.26, m	
	3			31.9
	4	29.3	2.97, m	
	5	54.8	2.86, m	
	6	49.7	2.62, q (7.2)	
	7	13.1	1.06, t (7.2)	
**Dimethoate**				
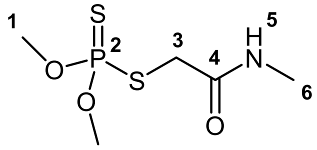	1	54.4	3.82, d (15.2)	
	2			97.5
	3	35.0	3.58, d (19.4)	
	4	170.4		
	5			
	6	25.9	2.78, s	
**Carbofuran**				
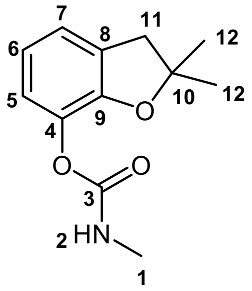	1	27.6	2.74, d (4.8)	
	2		5.84, brs	
	3	155.9		
	4	135.7		
	5	122.6	6.84, d (7.5)	
	6	120.6	6.78, t (7.5)	
	7	123.0	7.02, d (7.5)	
	8	130.7		
	9	151.5		
	10	89.1		
	11	43.3	3.06, s	
	12	27.3	1.44, s	
**Aminostigmine**				
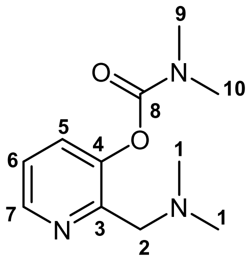	1	43.5	2.78, s	
	2	56.1	4.28, s	65.2
	3	144.0	-	
	4	148.7	-	
	5	132.4	7.66, dd (8.4; 1.4)	
	6	126.2	7.46, dd (8.4; 4.7)	
	7	147.2	8.48, dd (4.7; 1.4)	
	8	154.4	-	
	9	37.1	3.13, s	

	10	37.2	2.96, s	

Table S2. The ^{1}H, ^{13}C and ^{31}P NMR data of synthesis impurities in the amiton sample.

Compound	No.	δ_C, ppm	δ_H, ppm (*J*, Hz)	δ_P, ppm
**OP1**				
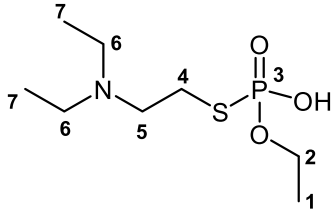	1	18.1	1.38, t (7.2)	
	2	68.4	4.25, m	
	3			32.7
	4	28.2	2.90, m	
	5	53.5	3.02, m	
	6	49.7	2.77, m	
	7	12.7	1.12, m	
**OP2**				
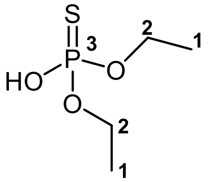	1	18.1	1.34, (7.2)	
	2	68.4	4.19, m	
	3			66.2
**OP3**				
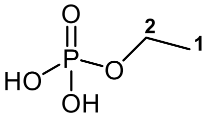	1	18.6	1.26, m	
	2	65.3	3.93, qui (7.1)	
	3			0.70
**OP4**				
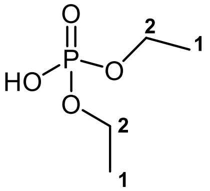	1	18.1	1.35, m	
	2	68.4	4.19, m	
	3			-0.49

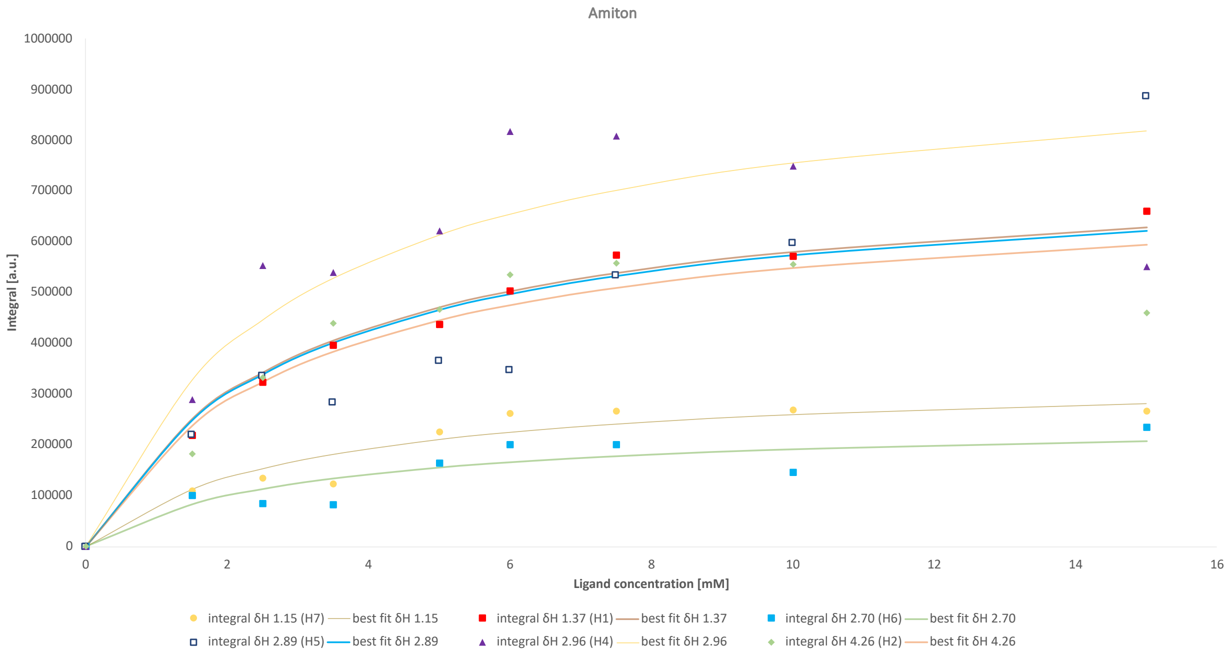

Figure S23. Amiton K_D determination. Individual peak intensities of ^{1}H NOE-pumping experiment (NOE mixing time 700 ms) as a function of ligand concentration and corresponding 1:1 binding model curves. Peak intensities were scaled according to number of protons within each moiety. Ligand concentrations 1.5-15.0 mM and BSA concentration 50 μM correspond to BSA:ligand molar ratios of 1:30, 1:50, 1:70, 1:100, 1:120, 1:150, 1:200 and 1:300 at 300 K. Solid lines represent the best fit curves of the data obtained using Microsoft Excel Solver.

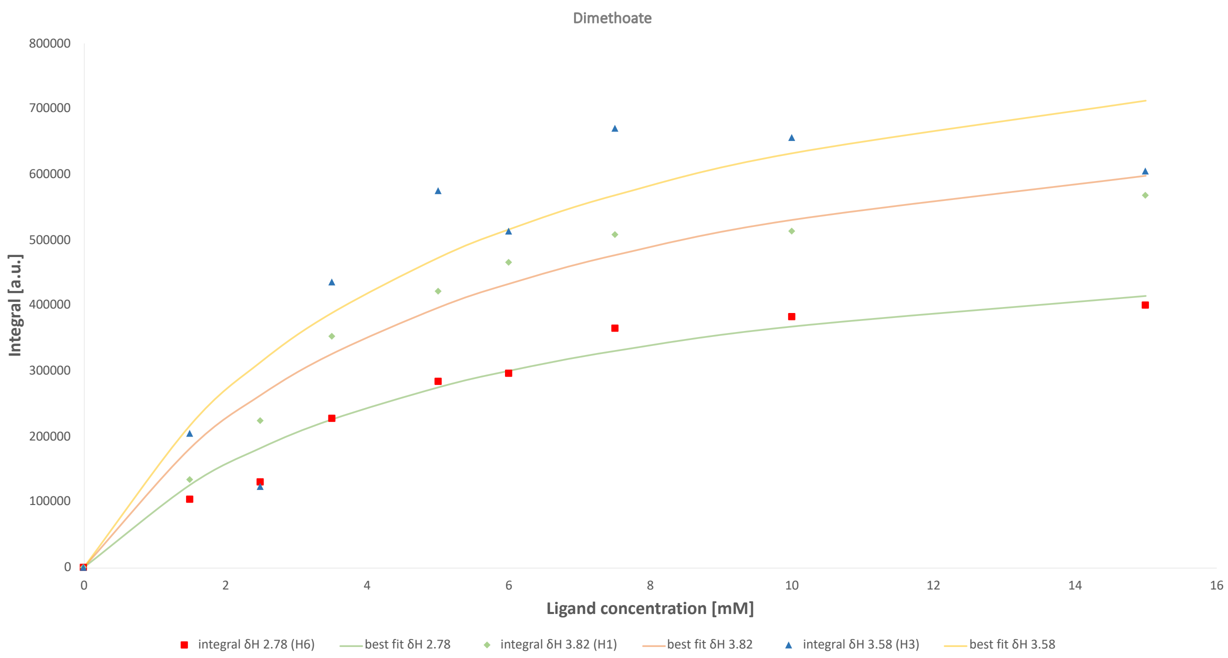

Figure S24. Dimethoate K_D determination. Individual peak intensities of ^{1}H NOE-pumping experiment (NOE mixing time 700 ms) as a function of ligand concentration and corresponding 1:1 binding model curves. Peak intensities were scaled according to number of protons within each moiety. Ligand concentrations 1.5-15.0 mM and BSA concentration 50 µM correspond to BSA:ligand molar ratios of 1:30, 1:50, 1:70, 1:100, 1:120, 1:150, 1:200 and 1:300 at 300 K. Solid lines represent the best fit curves of the data obtained using Microsoft Excel Solver.

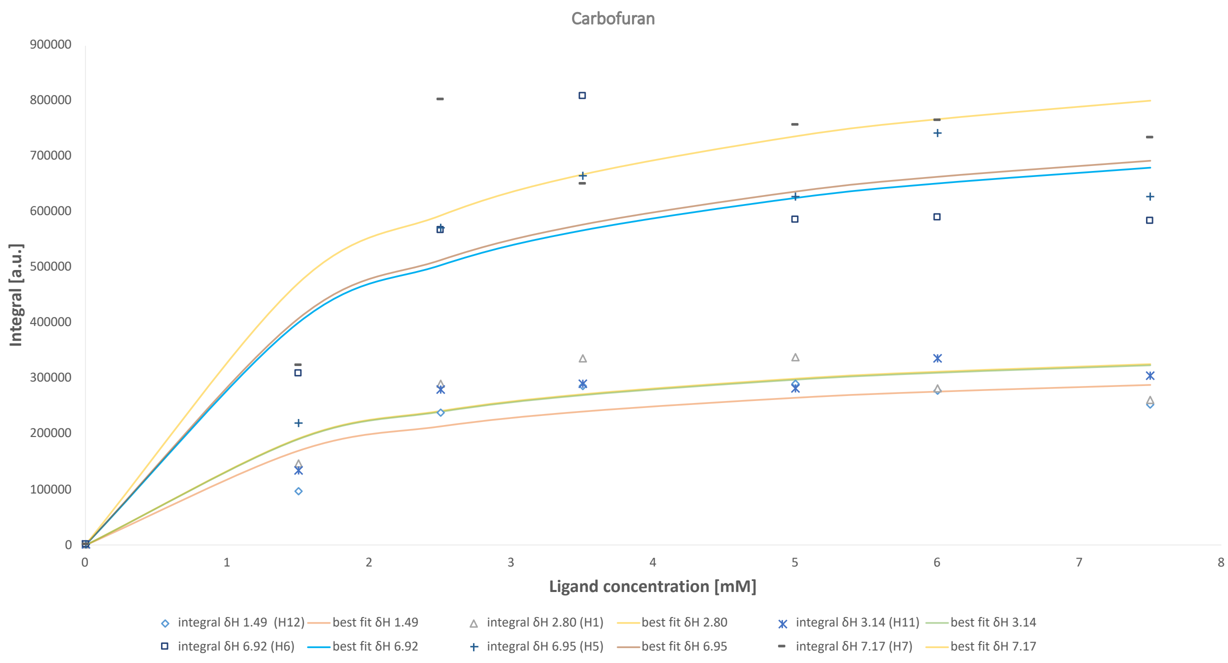

Figure S25. Carbofuran K_D determination. Individual peak intensities of ^1H NOE-pumping experiment (NOE mixing time 700 ms) as a function of ligand concentration and corresponding 1:1 binding model curves. Peak intensities were scaled according to number of protons within each moiety. Ligand concentrations 1.5-7.5 mM and BSA concentration 50 μM correspond to BSA:ligand molar ratios of 1:30, 1:50, 1:70, 1:100, 1:120, 1:150 at 300 K. Solid lines represent the best fit curves of the data obtained using Microsoft Excel Solver.

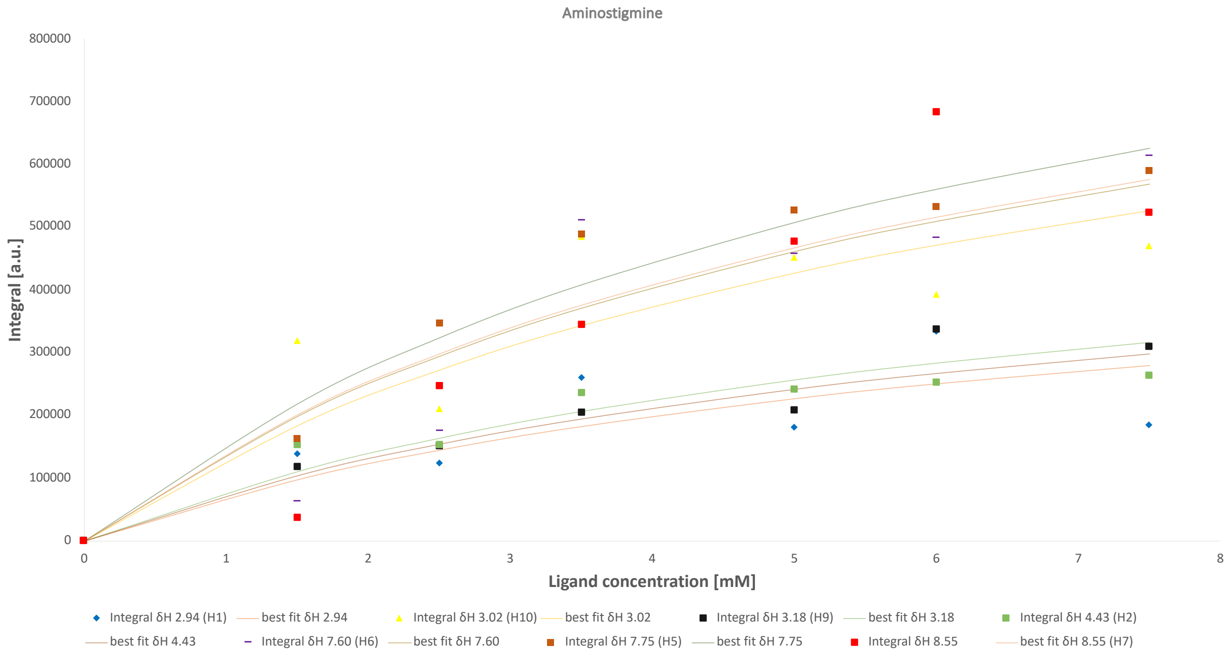

Figure S26. Aminostigmine K_D determination. Individual peak intensities of ^{1}H NOE-pumping experiment (NOE mixing time 700 ms) as a function of ligand concentration and corresponding 1:1 binding model curves. Peak intensities were scaled according to number of protons within each moiety. Ligand concentrations 1.5-15.0 mM and BSA concentration 50 µM correspond to BSA:ligand molar ratios of 1:30, 1:50, 1:70, 1:100, 1:120, 1:150 at 300 K. Solid lines represent the best fit curves of the data obtained using Microsoft Excel Solver.

Table S3. The obtained dissociation constants K_D for OP- and CM-BSA interactions.

	Amiton	*Dimethoate*	*Carbofuran*	*Aminostigmine*
K_D (M)	$3.0*10^{-3}$	$5.1*10^{-3}$	$1.6*10^{-3}$	$6.6*10^{-3}$

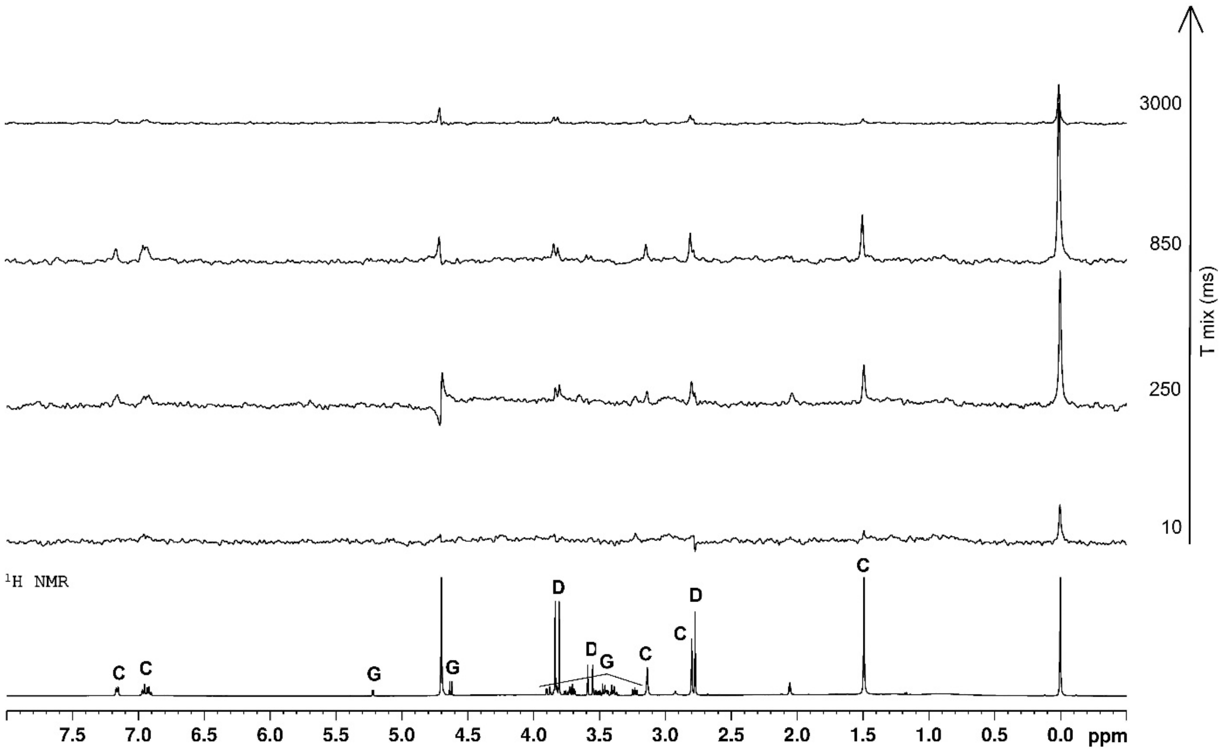

Figure S27. The ^{1}H NMR spectrum (bottom) with assignments for carbofuran ('C'), dimethoate ('D') and glucose ('G') and ^{1}H NOE pumping series with varying mixing times (10-3000 ms). Glucose was added in the solution as an internal control as it does not have affinity towards albumin and, thus can be utilized to monitor the performance of the NOE pumping operation via the absence of glucose signals in the ^{1}H NOE pumping spectra.

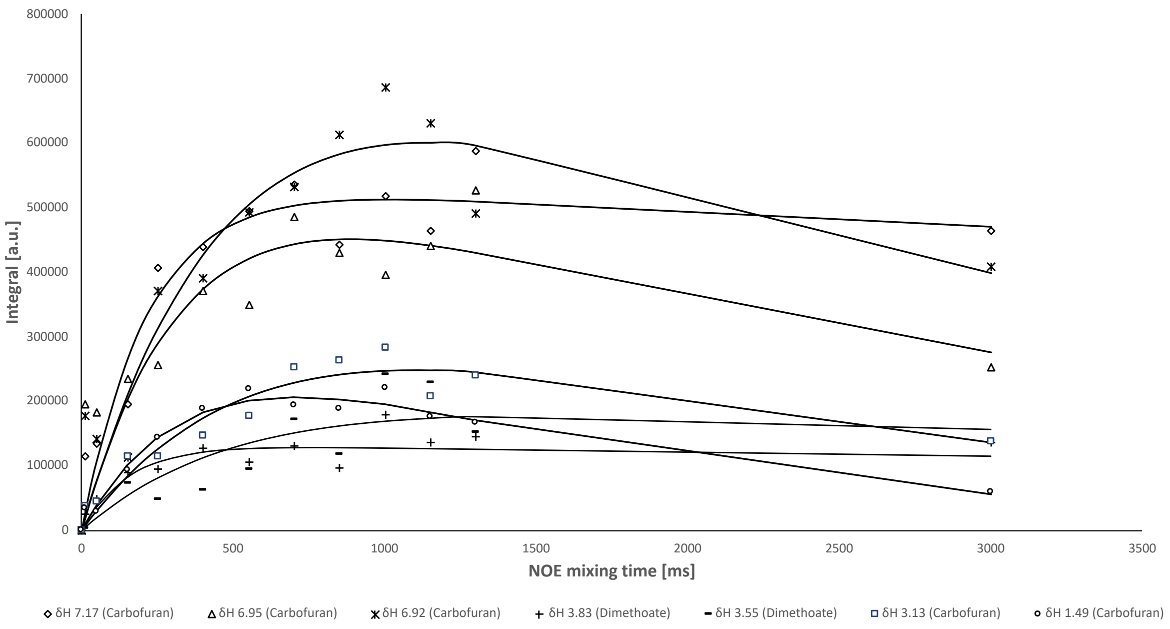

Figure S28. The NOE build-up evolution of carbofuran (◊ = H5, 7.17 ppm; Δ = H3, 6.95 ppm; ж = H4, 6.92 ppm; ☐ = H6, 3.13 ppm; ○ = H7, 1.49 ppm) and dimethoate (+ = H1, 3.82 ppm and ▬ = H2, 3.58 ppm) proton signals. Peak intensities were scaled according to number of protons within each moiety. Protons H_4 from dimethoate and H_1 from carbofuran were ignored, as we were not able to separate them due to chemical shift overlapping. Solid lines represent the obtained best fit of the data according to the transient NOE model. Best fit curves were created with Peak-o-mat software. Sample conditions: carbofuran = 5 mM, dimethoate = 5 mM and BSA = 50 µM; T = 300 K.

Pulse sequence. Pulse sequence used for ^{1}H NOE pumping experiments (ledbpgppr2s1d_T2filter, Bruker Avance NMR spectrometers)

```
;ledbpgppr2s1d_T2filter
;S. H., H.H. (modified and used for NOE-pumping)
;modified from avance-version (16/09/23)
;1D sequence for diffusion measurement using stimulated
;echo and LED
;using bipolar gradient pulses for diffusion
;using 2 spoil gradients
;with optional T2-filter just before the section using Carr-Purcell-Meiboom-Gill train
;activated by zgoptns -DT2_FILTER, duration controlled by loopcounter l4
;with gated presaturation during the delay d30 (within relaxation delay d1)
;BLKGRD now at the start of eddy current recovery delay Te (d21)
;for improving data quality when long Te-values are used in NOE-pumping
;
;D. Wu, A. Chen & C.S. Johnson Jr.,
; J. Magn. Reson. A 115, 260-264 (1995).
;A. Chen & M.J. Shapiro,
; JACS 120, 10258-10259 (1998).
;
;$CLASS=HighRes
;$DIM=1D
;$TYPE=
;$SUBTYPE=
;$COMMENT=


#include <Avance.incl>
#include <Grad.incl>
```

```
#include <Delay.incl>


"p2=p1*2"
"d11=30m"
"d31=d1-d30"
"DELTA1=d20-p1*2-p2-p30*2-d16*2-p19-d16"
"DELTA2=d21-p19-d16-4u-5m"
"DELTA3=d22-p2*0.5"


"acqt0=-p1*2/3.1416"


1 ze
2 d11 pl9:f1
d31
d30 cw:f1 ph29
4u do:f1
50u pl1:f1 UNBLKGRAD
p1 ph1

p30:gp6
d16
p2 ph1
p30:gp6*-1
d16
p1 ph2
p19:gp7
```

```
d16
DELTA1
p1 ph3
p30:gp6
d16
p2 ph1
p30:gp6*-1
d16
p1 ph4
p19:gp8
d16
5m
4u BLKGRAD
DELTA2

p1 ph5

# ifdef T2_FILTER
3 DELTA3
p2 ph6
DELTA3
DELTA3
p2 ph6
DELTA3
lo to 3 times l4
# else
1u
# endif /*T2_FILTER*/
```

```
go=2 ph31
d11 mc #0 to 2 F0(zd)
4u
Exit


ph1=0
ph2=0 0 2 2
ph3=0 0 0 0 2 2 2 2 1 1 1 1 3 3 3 3
ph4=0 2 0 2 2 0 2 0 1 3 1 3 3 1 3 1
ph5=0 0 0 0 2 2 2 2 1 1 1 1 3 3 3 3
ph6=1 1 1 1 1 1 1 1 2 2 2 2 2 2 2 2
ph29=0
ph31=0 2 2 0 2 0 0 2 3 1 1 3 1 3 3 1


;pl1 : f1 channel - power level for pulse (default)
;pl9 : f1 channel - power level for presaturation
;p1 : f1 channel - 90 degree high power pulse
;p2 : f1 channel - 180 degree high power pulse
;p19: gradient pulse (spoil gradient)
;p30: gradient pulse (little DELTA * 0.5)
;d1 : relaxation delay; 1-5 * T1
;d16: delay for gradient recovery
;d20: diffusion time (big DELTA)
;d21: eddy current delay (Te), can be used as NOE-mixing for NOE-pumping [5-5000 ms]
;d30: time of presaturation
```

;ns: 8 * n

;ds: 16 * m

;d22: fixed echo time to allow elimination of J-mod. effects

; d22 should be << 1/J ,but > (50 * P2) [0.5-2 msec]

;l4: loopcounter list for T2 filter [4 - 20]

;l4 can also have odd-values, as the basic cycle contains two echoes

;FnMODE: undefined

;zgoptns -DT2_FILTER

;T2-filter duration l4*4*d22

;use gradient ratio: gp 6 : gp 7 : gp 8

; diff : -17.13 : -13.17

;for z-only gradients:

;gpz6: 1-100%

;gpz7: -17.13% (spoil)

;gpz8: -13.17% (spoil)

;use gradient files:

;gpnam6: SMSQ10.100

;gpnam7: SMSQ10.100

;gpnam8: SMSQ10.100

;$Id: ledbpgp2s1d,v 1.9 2016/09/26 13:04:42 ber Exp $

Equations

Equation used to obtain the best fit of the data according to a simplified transient NOE model:

$$I(t_{mix}) = A_{SC} \times exp(-R_L\, t_{mix}) \times (1 - exp(-R_C\, t_{mix}))$$

$I(t_{mix})$ is the NOE-signal intensity in ^{1}H NOE-pumping experiment recorded using NOE mixing time t_{mix}. NOE-signal intensities are scaled according to the number of protons in the particular resonance prior to fitting process

A_{SC} is scaling factor

R_L is leakage rate constant (relaxation processes attenuating the signal)

R_C is cross relaxation rate constant (NOE build-up rate)

A three-parameter fit was used (A_{SC}, R_L and R_C). Fitting was performed using Peak-o-mat software (least squares minimization)

Equation used to obtain K_D values, 1:1 binding model:

$$I[L] = A_{SC} \times [L] / (K_D + [L])$$

$I[L]$ is the NOE signal intensity in ^{1}H NOE-pumping experiment recorded using a fixed mixing time and ligand concentration $[L]$. NOE-signal intensities are scaled according to the number of protons in the particular resonance prior to fitting process

A_{SC} is scaling factor

K_D is dissociation constant

A global K_D for each molecule was calculated. The titration data for each resonance was included in solving a single global K_D value for a molecule. For example, a system with three proton signals (1, 2 and 3) in ^{1}H NOE pumping spectrum results in three data point arrays. To solve global K_D (and thus obtain three curves) a four-parameter fitting is used K_D, A_{SC1}, A_{SC2} and A_{SC3} *i.e.* one global dissociation constant and three resonance specific scaling factors, respectively. The parameters were solved using GRG quasi-Newton non-linear regression algorithm (Lasdon, L. S.; Warren, A. D.; Jain, A.; Ratner, M.; Design and Testing of a

Generalized Reduced Gradient Code for Nonlinear Programming, *ACM Trans Math Softw* **1978**, *4*, 34–50.) in Microsoft Excel Solver routine.
